# Inhibition of caspase-1 or gasdermin-D enable caspase-8 activation in the Naip5/NLRC4/ASC inflammasome

**DOI:** 10.1371/journal.ppat.1006502

**Published:** 2017-08-03

**Authors:** Danielle P. A. Mascarenhas, Daiane M. Cerqueira, Marcelo S. F. Pereira, Fernanda V. S. Castanheira, Talita D. Fernandes, Graziele Z. Manin, Larissa D. Cunha, Dario S. Zamboni

**Affiliations:** Department of Cell Biology, School of Medicine of Ribeirão Preto, University of São Paulo. Ribeirão Preto, Brazil; Northwestern University Feinberg School of Medicine, UNITED STATES

## Abstract

*Legionella pneumophila* is a Gram-negative, flagellated bacterium that survives in phagocytes and causes Legionnaires’ disease. Upon infection of mammalian macrophages, cytosolic flagellin triggers the activation of Naip/NLRC4 inflammasome, which culminates in pyroptosis and restriction of bacterial replication. Although NLRC4 and caspase-1 participate in the same inflammasome, *Nlrc4*^*-/-*^ mice and their macrophages are more permissive to *L*. *pneumophila* replication compared with *Casp1/11*^*-/-*^. This feature supports the existence of a pathway that is NLRC4-dependent and caspase-1/11-independent. Here, we demonstrate that caspase-8 is recruited to the Naip5/NLRC4/ASC inflammasome in response to flagellin-positive bacteria. Accordingly, caspase-8 is activated in *Casp1/11*^*-/-*^ macrophages in a process dependent on flagellin, Naip5, NLRC4 and ASC. Silencing caspase-8 in *Casp1/11*^*-/-*^ cells culminated in macrophages that were as susceptible as *Nlrc4*^*-/-*^ for the restriction of *L*. *pneumophila* replication. Accordingly, macrophages and mice deficient in *Asc/Casp1/11*^*-/-*^ were more susceptible than *Casp1/11*^*-/-*^ and as susceptible as *Nlrc4*^*-/-*^ for the restriction of infection. Mechanistically, we found that caspase-8 activation triggers gasdermin-D-independent pore formation and cell death. Interestingly, caspase-8 is recruited to the Naip5/NLRC4/ASC inflammasome in wild-type macrophages, but it is only activated when caspase-1 or gasdermin-D is inhibited. Our data suggest that caspase-8 activation in the Naip5/NLRC4/ASC inflammasome enable induction of cell death when caspase-1 or gasdermin-D is suppressed.

## Author summary

*Legionella pneumophila* is the causative agent of Legionnaires’ disease, an atypical pneumophila that affects people worldwide. Besides the clinical importance, *L*. *pneumophila* is a very useful model of pathogenic bacteria for investigation of the interactions of innate immune cells with bacterial pathogens. Studies using *L*. *pneumophila* demonstrated that Naip5 and NLRC4 activate caspase-1 and this inflammasome is activated by bacterial flagellin. However, macrophages and mice deficient in NLRC4 are more susceptible for *L*. *pneumophila* replication than those deficient in caspase-1, indicating that the flagellin/Naip5/NLRC4 inflammasome triggers responses that are independent on caspase-1. Here, we used *L*. *pneumophila* to investigate this novel pathway and found that caspase-8 interacts with NLRC4 in a process that is dependent on ASC and independent of caspase-1 and caspase-11. Although caspase-8 is recruited to the Naip5/NLRC4/ASC inflammasome, it is only activated when caspase-1 or gasdermin-D is inhibited. Our data suggest that caspase-8 activation in the Naip5/NLRC4/ASC inflammasome may favor host responses during infections against pathogens that inhibit components of the pyroptotic cell death including caspase-1 and gasdermin-D.

## Introduction

*Legionella pneumophila* is the causative agent of Legionnaires’ disease. It was identified for the first time in 1976, after an atypical pneumonia affected the participants of the American Legion Convention in Philadelphia, United States [1]. After isolation, *L*. *pneumophila* were characterized as Gram-negative, flagellated, intracellular facultative bacteria [2,3]. The species of *Legionella* were found mainly in freshwater and soil environments, including lakes and irrigation systems [4]. Infection of humans occurs upon inhalation of water droplets derived from these environments containing *Legionella* [5]. After inhalation, *L*. *pneumophila* can subvert the normal vesicle traffic within alveolar macrophages and form LCV (*Legionella*-containing vacuoles), a process that is mediated by the injection of hundreds of bacterial effectors through a type IV secretion system called Dot/Icm [6–9]. During its evolution, *L*. *pneumophila* were selected based on their replication in protozoa but not in humans, which are accidental hosts [10]. Consequently, *L*. *pneumophila* can be recognized by many innate immune receptors in mammalian cells, including proteins from the family of the nucleotide-binding domain and leucine-rich repeat-containing proteins (NLRs). These characteristics make *L*. *pneumophila* an excellent model for the study of innate immunity, including intracellular signaling pathways and inflammasomes.

The major inflammasome that leads to the restriction of *Legionella* replication in macrophages is Naip5/NLRC4. This pathway was discovered in mouse cells upon observations that macrophages from the A/J mouse strain, but not cells from other mice strains, are susceptible to *L*. *pneumophila* replication [11]. The resistance was mapped to the *Lgn1* locus, which encodes several copies of *Naip* genes, including *Naip5* (*Birc1e*), which is the gene responsible for resistance [12–16]. Successful lines of investigation culminated in the demonstration that Naip5 recognizes bacterial flagellin and interacts with NLRC4 for caspase-1 activation and the restriction of bacterial replication [17–20]. This platform was named the Naip5/NLRC4 inflammasome and triggers pore formation and pyroptosis, which has been considered one of the most important mechanisms for the restriction of intracellular pathogen replication via inflammasomes [21–24]. Host cell death via pyroptosis eliminates intracellular parasite replication and traps intracellular microbes in pyroptotic cells, facilitating microbial destruction by additional phagocytes [23,25–29]. Pyroptosis occurs concomitantly with the secretion of inflammatory cytokines such as IL-1β and IL-18, a process that requires the adaptor molecule ASC and the formation of NLRC4/ASC puncta [20,30]. ASC also functions as an adaptor protein for other inflammasomes, including AIM2 and NLRP3, which triggers the processing of caspase-1 and caspase-8 [21,31–34]. Of note, Naip5/NLRC4 appears to be the only inflammasome required for the restriction of *L*. *pneumophila* replication. Macrophages that are deficient in NLRP3 or AIM2 can efficiently restrict *L*. *pneumophila* replication [20,21,23,35]. However, the participation of ASC in the resistance of *L*. *pneumophila* infection is controversial. In murine macrophages, ASC is dispensable for the induction of pyroptosis and the restriction of bacterial replication [20,21]. By contrast, experiments performed with human monocytes indicate that ASC silencing leads to an increase in bacterial replication [36,37]. Thus, the role of ASC in the restriction of *L*. *pneumophila* replication is still unclear.

We have previously demonstrated the existence of a pathway that is dependent on flagellin and NLRC4 but independent of caspase-1 [38]. Here, we used macrophages and *Casp1/11*^*-/-*^ mice to systematically assess this pathway. By searching for additional components that operate in the NLRC4 inflammasome independently of caspase-1/11, we found that caspase-8 interacts with NLRC4 in a process that is dependent on ASC. This pathway effectively accounts for resistance to infection in macrophages and in vivo when caspase-1 is absent. In wild-type cells, caspase-8 is recruited to the Naip5/NLRC4/ASC/caspase-1 inflammasome, but is not activated. Caspase-8 activation in this platform only occurs when caspase-1 or gasdermin-D is inhibited, suggesting that this pathway may be important when pyroptosis is inhibited.

## Results

### Restriction of *L*. *pneumophila* replication in BMDMs is flagellin/NLRC4-dependent, ASC-independent and partially caspase-1/11-dependent

We have previously demonstrated that activation of the flagellin/NLRC4 inflammasome triggers caspase-1-dependent and independent responses to restrict *Legionella* replication in macrophages and in mouse lungs [38]. However, the caspase-1-independent mechanisms underlying this pathway are unknown. To further characterize this pathway, we performed growth curves using high and very low multiplicity of infections (MOIs) in bone marrow-derived macrophages (BMDMs). Macrophages were infected with wild-type *L*. *pneumophila* in the JR32 background (WT Lp) and the isogenic mutants *flaA*^*-*^ and *fliI*^*-*^. FliI is an ATPase that is required for the secretion of flagellin through the flagellar apparatus [39]. Consequently, *fliI*^-^ mutants express flagellin but are non-motile and non-flagellated, making them an appropriate control for *flaA*^*-*^ mutants for investigations related to the role of flagellin. We found that BMDMs from C57BL/6 and *Asc*^-/-^ mice fully restrict the replication of WT Lp and *fliI*^*-*^ bacteria at low and high MOIs. In contrast, *Nlrc4*^*-/-*^ cells are permissive and *Casp1/11*^-/-^ cells are partially restrictive (S1 Fig). Bacterial mutants for flagellin bypass NLRC4-mediated growth restriction and replicate in all macrophages as previously described [17–19,40]. These data support previous reports showing that ASC is not required for the restriction of *L*. *pneumophila* replication in the presence of caspase-1/11 [20,21]. In addition, these data further support our previous assertion that flagellin triggers an uncharacterized pathway that is dependent on NLRC4 and independent of caspase-1 and caspase-11 [38]. We decided to use BMDMs from *Casp1/11*^-/-^ mice to further investigate this NLRC4-dependent and caspase-1/11-independent pathway.

**Fig 1:**
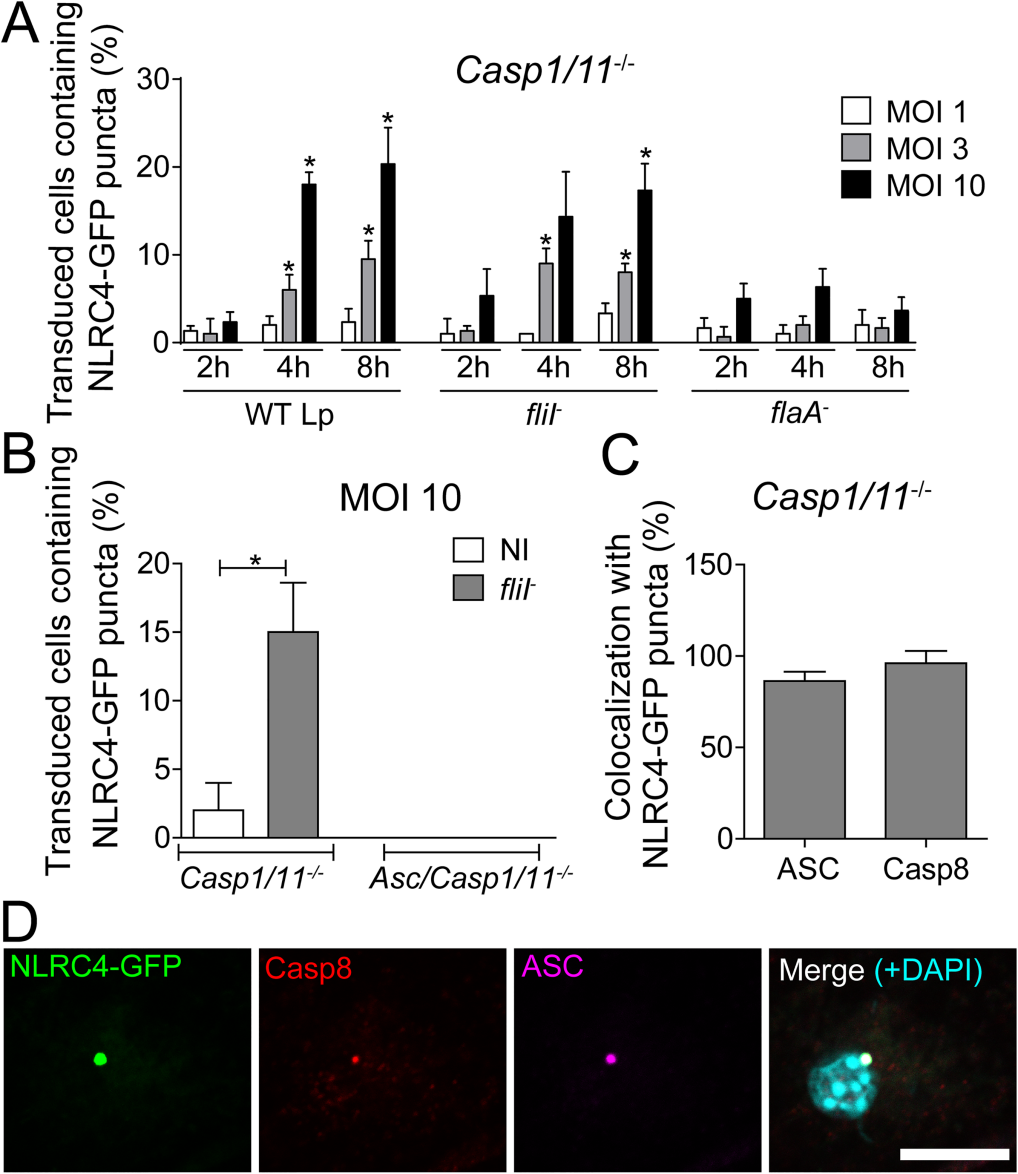
*Legionella* triggers the formation of NLRC4/caspase-8 puncta in a process that is dependent on ASC and flagellin and independent of caspase-1/11. Bone marrow-derived macrophages (BMDMs) obtained from *Casp1/11*^*-/-*^ and *Asc/Casp1/11*^*-/-*^ mice were transduced with retrovirus encoding NLRC4-GFP. Cells were infected with wild-type *L*. *pneumophila* (WT Lp), motility-deficient mutants expressing flagellin (*fliI*^-^) or with flagellin-deficient mutants (*flaA*^*-*^) at a MOI of 1, 3 or 10. (A) After 2, 4 and 8 hours of infection, the cells were fixed, and the percentage of transduced cells containing NLRC4-GFP puncta were determined using an epifluorescence microscope. *, *P*<0.05 compared with BMDMs infected with *flaA*^*-*^, Student’s *t* test. (B-D) Transduced cells were infected with *fliI*^*-*^ (MOI 10) and fixed after 8 hours of infection. (B) The percentages of transduced *Casp1/11*^*-/-*^ and *Asc/Casp1/11*^*-/-*^ cells containing NLRC4-GFP puncta were determined. (C) The percentages of NLRC4-GFP puncta that colocalized with ASC and caspase-8 were determined. (D) Representative images of a transduced BMDM infected with *fliI*^-^ at a MOI of 10. The cultures were stained with anti-caspase-8 (red) and anti-ASC (purple). The cell nuclei were stained with DAPI (cyan); NLRC4-GFP is shown in green. The images show the colocalization of NLRC4-GFP, ASC and caspase-8 in *Casp1/11*^*-/-*^ BMDMs. The images were acquired by multiphoton microscopy using a 63x oil immersion objective and analyzed using ImageJ Software. Scale bar, 10μm. Data show the average ± SD of triplicate wells. NI, uninfected. Data are presented for one representative experiment of five (A) and two (B-D) experiments with similar results.

### Flagellated *L*. *pneumophila* triggers NLRC4 puncta that associate with caspase-8 in a process that is ASC-dependent

**Fig 2:**
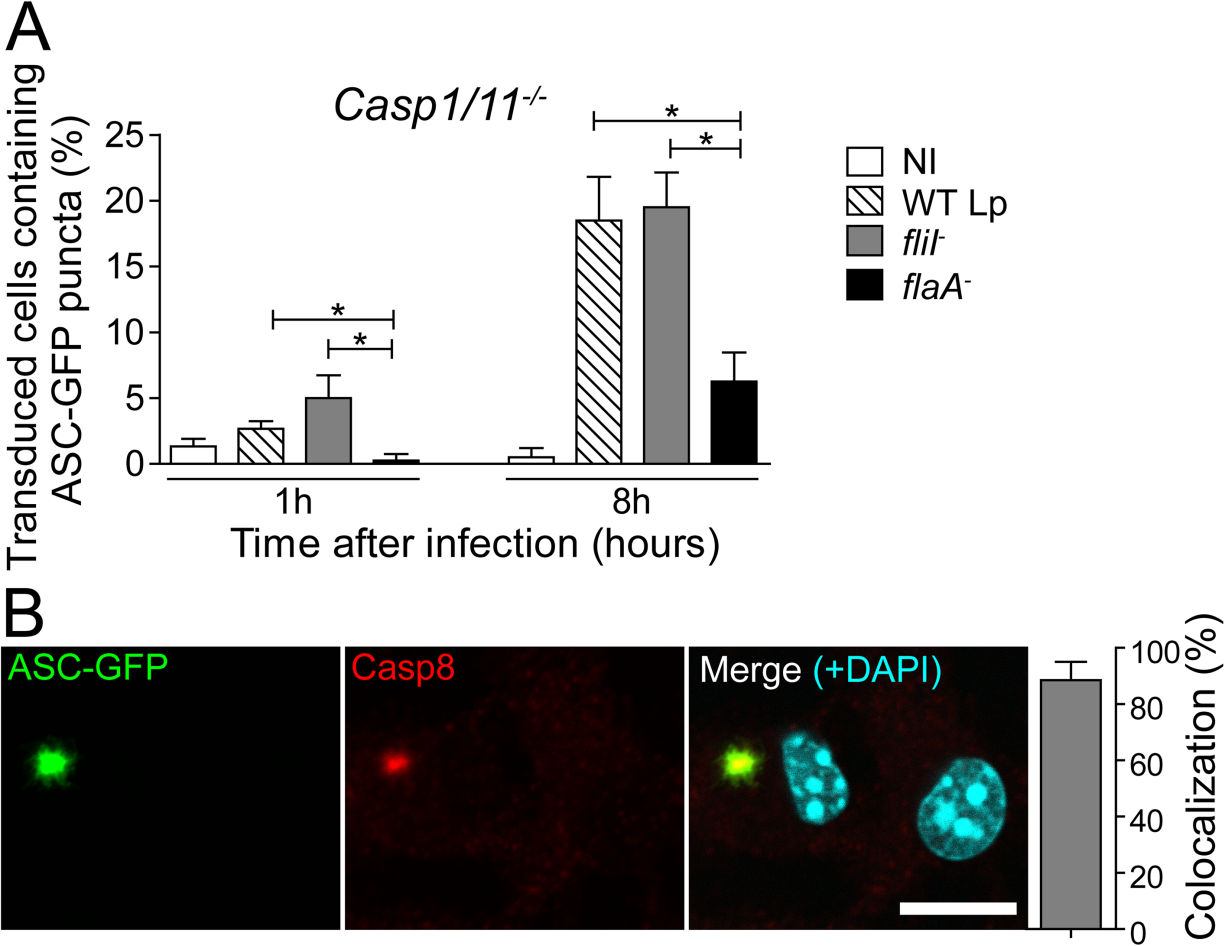
ASC puncta are induced in response to flagellated bacteria and colocalize with caspase-8. Bone marrow-derived macrophages (BMDMs) from *Casp1/11*^*-/-*^ mice were transduced with retrovirus encoding ASC-GFP. (A) Cells were infected with wild-type *L*. *pneumophila* (WT Lp), motility-deficient mutants expressing flagellin (*fliI*^-^) or with flagellin-deficient mutants (*flaA*^*-*^) at a MOI of 10. After 1 or 8 hours of infection, the cells were fixed, and the number of transduced cells containing ASC-GFP puncta was determined using an epifluorescence microscope. (B) Representative images of a transduced BMDM infected with *fliI*^-^ at a MOI of 10. The cultures were stained with anti-caspase-8 (red), cell nuclei were stained with DAPI (cyan) and ASC-GFP is shown in green. The images show the colocalization between ASC-GFP and caspase-8 in *Casp1/11*^*-/-*^ BMDMs infected for 8 hours. The images were acquired by multiphoton microscopy with a 63x oil immersion objective and analyzed using ImageJ Software. Scale bar, 10μm. Data show the average ± SD of triplicate wells. *, *P*<0.05, Student’s *t* test. NI, uninfected. Data are presented for one representative experiment of three (A) and two (B) experiments with similar results.

The transduction of BMDMs with a retrovirus encoding NLRC4 fused to GFP allows the visualization of NLRC4 puncta in the cytoplasm of macrophages infected with flagellated bacteria [30]. Here, we used this retroviral system to investigate the formation of the NLRC4 inflammasome in the absence of caspase-1/11. BMDMs from *Casp1/11*^*-/-*^ mice were transduced with NLRC4-GFP and infected with WT Lp, *fliI*^*-*^ and *flaA*^*-*^ at different MOIs and time points. We found that WT Lp and *fliI*^-^ triggered the formation of NLRC4 puncta in the absence of caspase-1/11 (Fig 1A). The formation of NLRC4 puncta was influenced by the MOI and significantly diminished in response to *flaA*^-^ bacteria (Fig 1A). Next, we evaluated the requirement of ASC for the formation of NLRC4 puncta in the absence of caspase-1/11. We constructed a mouse that was deficient in ASC and caspase-1/11 and found that whereas the formation of NLRC4 puncta occurred in the absence of caspase-1/11, ASC was essential for formation of the NLRC4 puncta (Fig 1B). These data are in agreement with previous findings indicating that ASC is critical for the nucleation of several inflammasomes, including AIM2, NLRP3 and NLRC4 [30,32,33,41–46]. Our results confirm that ASC is essential to NLRC4 puncta formation formed in the absence of caspase-1/11. Next, we used this NLRC4-GFP system to identify additional components of the NLRC4 inflammasome that operates in the absence of caspase-1/11. Non-inflammatory caspases have been previously shown to participate in the assembly of inflammasomes and to interact with ASC, including caspase-3, caspase-7 and caspase-8 [32–34,37,44,46–51]. Thus, we transduced *Casp1/11*^*-/-*^ macrophages with a retrovirus encoding NLRC4-GFP and evaluated the colocalization of NLRC4 with these caspases. In this experiment, we used the pan-caspase inhibitor Z-VAD to block caspase activation and to visualize puncta formation. We did not detect significant numbers of NLRC4 or ASC puncta containing caspase-3 and caspase-7 (S2 Fig). In contrast, caspase-8 and ASC was present in more than 90% of the NLRC4 puncta (Fig 1C and S2 Fig). These data are in agreement with our findings indicating that ASC is required for NLRC4 puncta formation, accordingly, endogenous ASC colocalizes with NLRC4 and caspase-8 in the same puncta (Fig 1D). To evaluate the participation of caspase-8 in the NLRC4 inflammasome, we transduced BMDMs from *Casp1/11*^*-/-*^ mice with a retrovirus encoding ASC fused to GFP (ASC-GFP) and analyzed ASC puncta colocalization with caspase-8. We found that ASC puncta formed readily after the infection and that this process occurred in response to WT Lp and *fliI*^-^ but not *flaA*^*-*^ (Fig 2A). After 8 hours of infection, the formation of ASC puncta was partially dependent on flagellin (Fig 2A). We stained caspase-8 in macrophages transduced with retrovirus encoding ASC-GFP and found that caspase-8 colocalized with ASC puncta in response to infection with flagellated bacteria (Fig 2B). Collectively, these results indicate that flagellin triggers the assembly of an inflammasome composed of NLRC4 and ASC, which colocalizes with caspase-8.

The double-stranded DNA sensor AIM2 is known to recruit ASC to trigger puncta formation in response to infection, leading to caspase-1 activation and IL-1β and IL-18 release [31,52–55]. The role of AIM2 inflammasome in the recognition of *L*. *pneumophila* has been demonstrated using *sdhA*^*-*^ deficient bacteria. In the absence of SdhA, bacteria do not maintain vacuole integrity and localize in the macrophage cytoplasm, triggering activation of the AIM2 inflammasome [56,57]. In addition, the AIM2 inflammasome has been shown to trigger caspase-8 activation independently of caspase-1 [33,34,58]. Thus, we investigated whether AIM2 is present in the NLRC4/ASC/caspase-8 inflammasome that is formed in response to flagellin-positive *L*. *pneumophila*. We stained AIM2 in macrophages transduced with retrovirus encoding NLRC4-GFP and found no AIM2 in the NLRC4 puncta (S3A Fig). Moreover, we generated *Aim2/Casp1/11*^*-/-*^ mice and found that AIM2 was dispensable for the formation of NLRC4 puncta in response to flagellin-positive bacteria (S3B Fig).

### The NLRC4/ASC/caspase-8 inflammasome accounts for flagellin-dependent restriction of *Legionella* replication in macrophages in the absence of caspase-1/11

**Fig 3:**
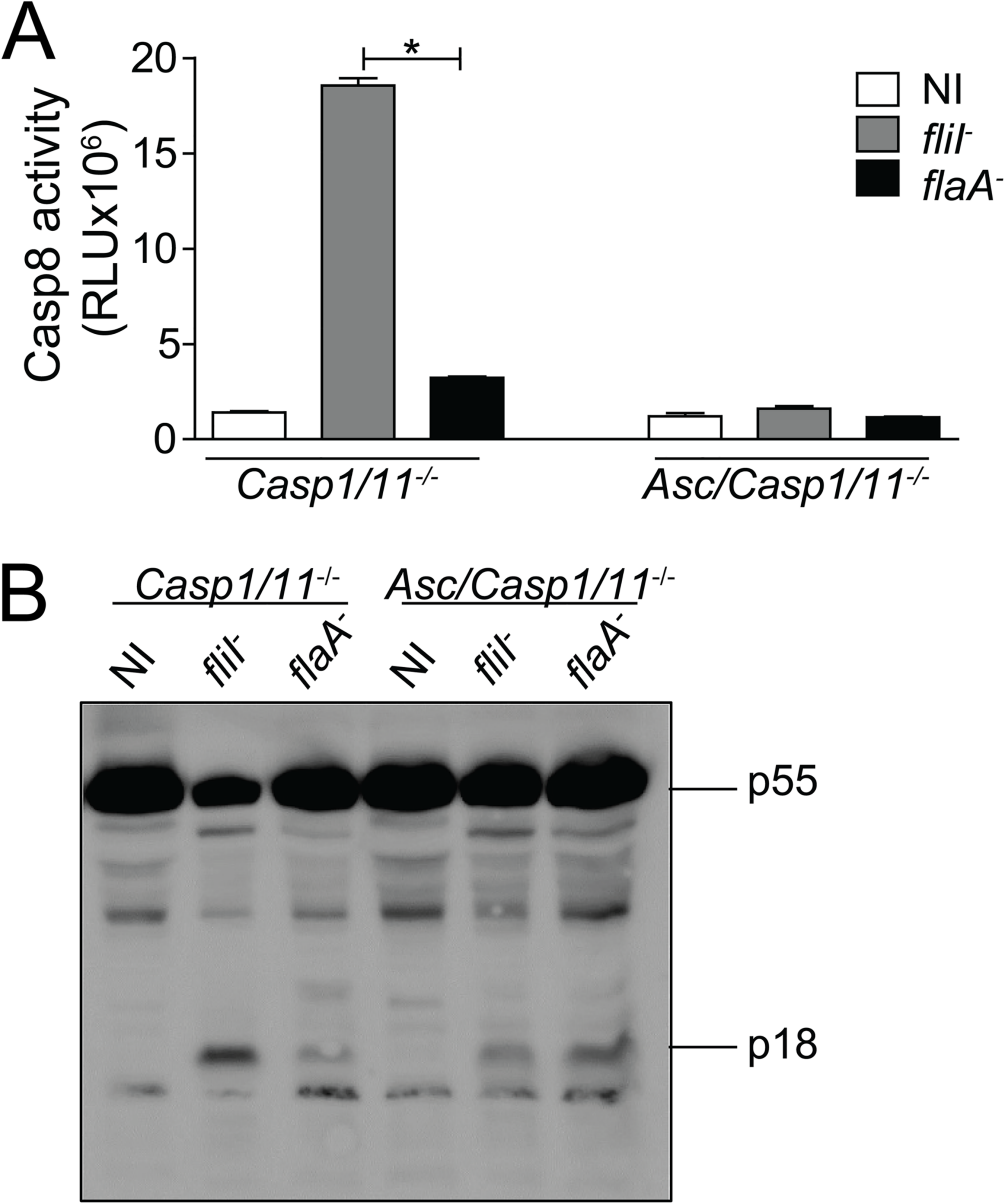
Caspase-8 is activated in response to flagellated bacteria in a process that is ASC-dependent and caspase-1/11-independent. Bone marrow-derived macrophages **(**BMDMs) from *Casp1/11*^*-/-*^ and *Asc/Casp1/11*^*-/-*^ mice were infected with motility-deficient *L*. *pneumophila* mutants expressing flagellin (*fliI*^-^) or with flagellin-deficient bacteria (*flaA*^*-*^) at a MOI of 10 for 8 hours. The activity of caspase-8 was measured using the Caspase-8 Glo Assay (A) or by western blot analysis (B). The pro-caspase-8 p55 and the processed form of caspase-8 p18 are indicated. Data show the average ± SD of triplicate wells. *, *P*<0.05, Student’s *t* test. RLU, relative luminescence units; NI, uninfected. Data are presented for one representative experiment of three (A) and two (B) experiments with similar results.

Our data are consistent with the hypothesis that caspase-8 is a part of the inflammasome composed of NLRC4 and ASC. Thus, we investigated whether caspase-8 is activated during infection. We found that caspase-8 was strongly activated in *Casp1/11*^*-/-*^ BMDMs in response to *fliI*^-^ but not *flaA*^-^ bacteria (Fig 3A). In agreement with the requirement of ASC for the assembly of the NLRC4/ASC/caspase-8 inflammasome, we found that caspase-8 activation was abolished in *Asc/Casp1/11*^*-/-*^ cells (Fig 3A). Caspase-8 activation occurred normally in *Aim2/Casp1/11*^*-/-*^ cells, indicating that AIM2 was not involved in the activation of caspase-8 through the flagellin/NLRC4/ASC inflammasome (S4 Fig). We also evaluated caspase-8 activation by western blot analysis by measuring the cleavage of p55 and the production of p18 isoforms. We found that flagellated bacteria triggered caspase-8 activation in *Casp1/11*^*-/-*^ but not in *Asc/Casp1/11*^*-/-*^ cells. This phenomenon was evident by the reduction in p55 and increased production of p18 in *Casp1/11*^*-/-*^ BMDMs infected with *fliI*^*-*^ but not *flaA*^*-*^ bacteria (Fig 3B).

**Fig 4:**
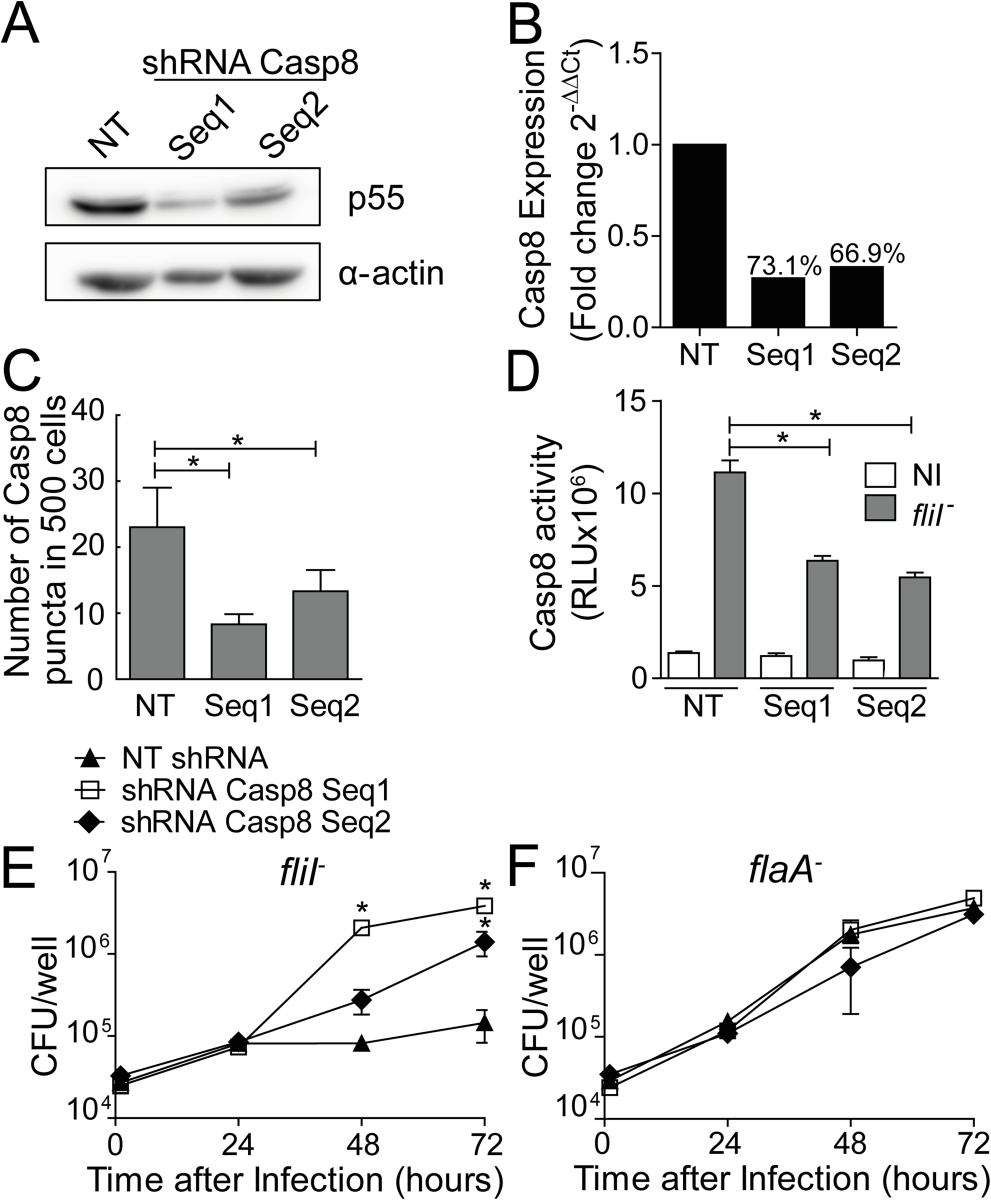
Caspase-8 is important for NLRC4-mediated restriction *of L*. *pneumophila* replication independently of caspase-1/11. Bone marrow-derived macrophages (BMDMs) generated from *Casp1/11*^*-/-*^ mice were transduced with a retrovirus encoding shRNA sequences to target caspase-8 (Seq1, Seq2) and a non-target shRNA sequence (NT). (A) The silencing was confirmed by western blot analysis and Real Time qPCR. (A) Cell lysates were separated by SDS-PAGE, blotted and probed with anti-caspase-8 (pro-caspase-8 p55) and anti-α-actin. (B) Quantification of the *casp8* gene expression by Real Time qPCR. *Actin beta* gene was used as a control for normalization of expression levels. The number above the bars indicates the percentage of silencing compared to the NT sequence. (C-D) The transduced *Casp1/11*^*-/-*^ BMDMs were infected with motility-deficient *L*. *pneumophila* mutants expressing flagellin (*fliI*^-^) at a MOI of 10. After 8 hours the cells were fixed, the number of caspase-8 puncta was quantified using an epifluorescence microscope (C) and the activity of caspase-8 was measured using the Caspase-8 Glo Assay (D). (E-F) The transduced *Casp1/11*^*-/-*^ BMDMs were infected with *fliI*^*-*^ (E) and *flaA*^*-*^ (F) at a MOI of 10 to evaluate bacterial replication. The cells were incubated for 24, 48 and 72 hours for CFU determination. Data show the average ± SD of triplicate wells. *P<0.05, compared with NT. (C-D) Student’s *t* test, (E-F) ANOVA. RLU, relative luminescence units; NI, uninfected. Data are presented for one representative experiment of three experiments with similar results.

Next, we evaluated the participation of caspase-8 in caspase-1/11-independent restriction of *L*. *pneumophila* replication in macrophages, a process that was dependent on flagellin and NLRC4. Endogenous caspase-8 was silenced in *Casp1/11*^*-/-*^ BMDMs using two independent retrovirus encoding shRNA to target caspase-8. A non-target sequence was used as a control (NT). By western blotting, we detected reduced caspase-8 expression in *Casp1/11*^*-/-*^ transduced cells. The shRNA Casp8 Seq1 was more efficient than Seq2 for silencing caspase-8 as determined by western blot (Fig 4A and S5 Fig) and RT-PCR (Fig 4B). Importantly, complete silencing of caspase-8 cannot be achieved because caspase-8 expression is required for macrophage survival [59,60]. Nonetheless, using the described silencing conditions, we did not detect signs of cell death or LDH in the supernatant of the transduced macrophages. To evaluate the efficiency of caspase-8 silencing, we quantified caspase-8-containing puncta formation and caspase-8 activation in macrophages infected with flagellated *L*. *pneumophila*. We found that the frequency of puncta containing caspase-8 and caspase-8 activation was reduced in caspase-8-silenced cells (Fig 4C and 4D). Next, we evaluated the effect of caspase-8 for the restriction of *L*. *pneumophila* replication in *Casp1/11*^*-/-*^ BMDMs. We found that silencing caspase-8 culminated in increased replication of *fliI*^*-*^ but not *flaA*^-^ bacteria (Fig 4E and 4F). These data indicated that caspase-8 contributed to the restriction of bacterial replication in a process that was dependent on flagellin, supporting the hypothesis that caspase-8 functionally participates in responses that are NLRC4/ASC-dependent and caspase-1/11-independent.

### ASC is required for NLRC4/caspase-8-mediated restriction of *Legionella* replication in the absence of caspase-1/11

**Fig 5:**
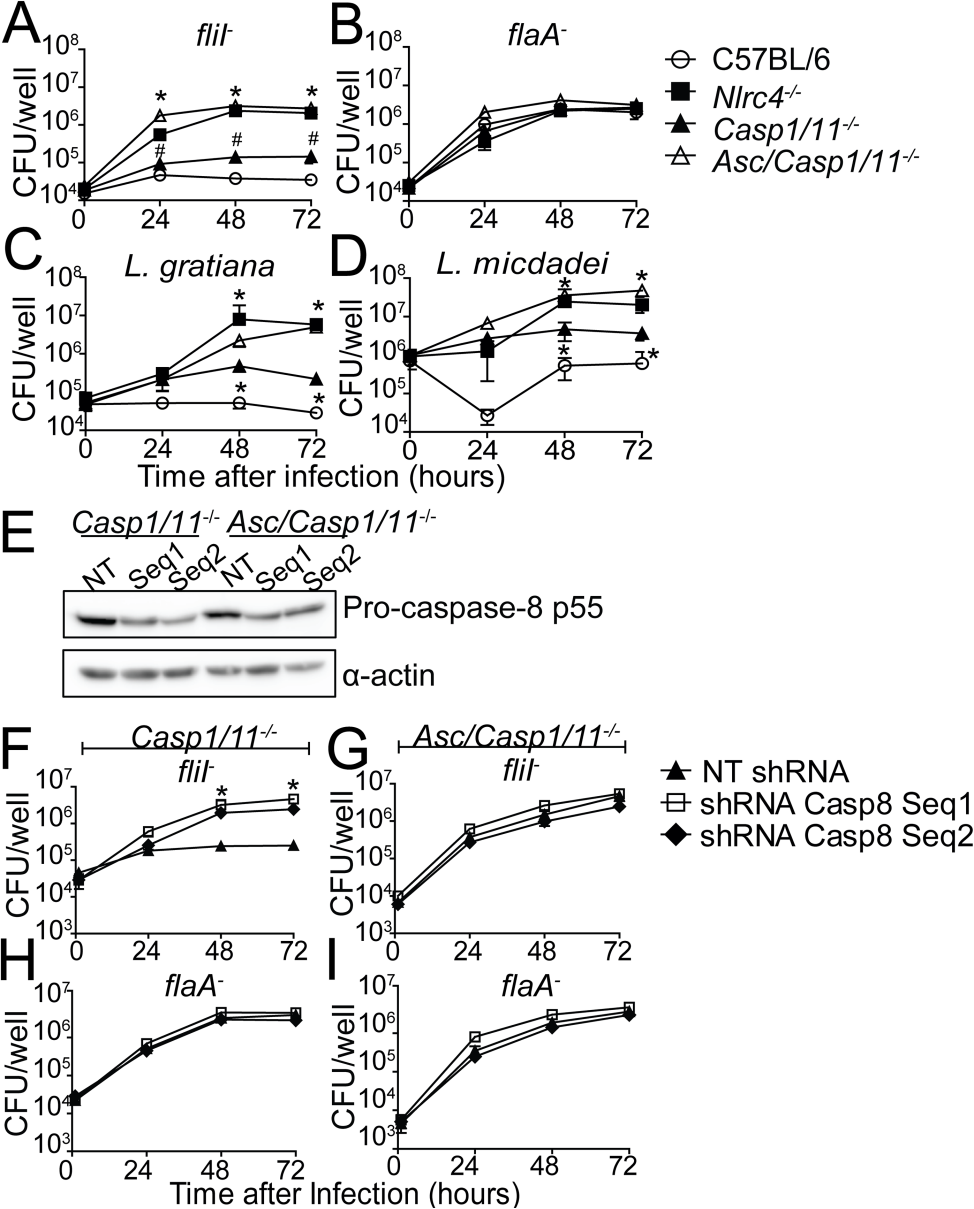
ASC is important for NLRC4/caspase-8-mediated restriction *of L*. *pneumophila* replication independently of caspase-1/11. (A-D) Bone marrow-derived macrophages (BMDMs) from C57BL/6 (open circles), *Nlrc4*^*-/-*^ (closed squares), *Casp1/11*^*-/-*^ (closed triangles) and *Asc/Casp1/11*^*-/-*^ (open triangles) mice were infected with motility-deficient mutants expressing flagellin (*fliI*^-^, A), with flagellin-deficient mutants (*flaA*^*-*^, B), *L*. *gratiana* (C) or with *L*. *micdadei* (D) at a MOI of 10. The cells were incubated for 24, 48 and 72 hours for CFU determination. Data show the average ± SD of triplicate wells. *, *P*<0.05 compared with *Casp-1/11*^*-/-*^ BMDMs. ^#^, *P*<0.05 compared with C57BL/6 BMDMs, ANOVA. (E-I) BMDMs from *Casp1/11*^*-/-*^ and *Asc/Casp1/11*^*-/-*^ mice were transduced with a retrovirus encoding shRNA sequences to target caspase-8 (Seq1, Seq2) and a non-target shRNA sequence (NT). (E) Caspase-8 silencing was confirmed by western blot analysis. Cell lysates were separated by SDS-PAGE, blotted and probed with anti-caspase-8 (pro-caspase-8 p55) and anti-α-actin. (F-I) Transduced *Casp1/11*^*-/-*^ (F, H) and *Asc/Casp1/11*^*-/-*^ (G, I) BMDMs were infected with *fliI*^*-*^ (F, G) or *flaA*^*-*^ (H, I) at a MOI of 10 and incubated for 24, 48 and 72 hours for CFU determination. Data show the average ± SD of triplicate wells. *, *P*<0.05 compared with NT shRNA, ANOVA. NT, non-target shRNA. Data are presented for one representative experiment of four (A), two (B-D) and one (F-I) experiments performed with similar results.

Our data indicate that caspase-8 is part of the NLRC4/ASC inflammasome and that ASC is essential for the assembly of this inflammasome. Thus, we reasoned that in the absence of ASC, the NLRC4/ASC/caspase-8 inflammasome would not be functional. If this hypothesis is correct, *Asc/Casp1/11*^*-/-*^ macrophages should be more permissive than *Casp1/11*^*-/-*^ and as permissive as *Nlrc4*^*-/-*^. We infected C57BL/6, *Casp1/11*^*-/-*^, *Asc/Casp1/11*^*-/-*^ and *Nlrc4*^*-/-*^ BMDMs with *fliI*^*-*^ and *flaA*^*-*^, and evaluated bacterial replication after 24, 48 and 72 hours. Using flagellin-positive bacteria, we confirmed that C57BL/6 BMDMs were restrictive to bacterial growth, *Nlrc4*^*-/-*^ were permissive and *Casp1/11*^*-/-*^ were partially restrictive (Fig 5A). Importantly, *Asc/Casp1/11*^*-/-*^ cells were highly permissive and phenocopied the *Nlrc4*^*-/-*^ cells (Fig 5A). As predicted, flagellin mutants bypassed the NLRC4 and replicated in all macrophages evaluated (Fig 5B). These data further confirmed that *Casp1/11*^*-/-*^ cells were more restrictive than *Nlrc4*^*-/-*^ macrophages, possibly due to the presence of the NLRC4/ASC/caspase-8 inflammasome. We also used non-pneumophila species to compare bacterial replication in *Nlrc4*^*-/-*^ and *Asc/Casp1/11*^*-/-*^ macrophages. Infections performed with *L*. *gratiana* and *L*. *micdadei* indicated that *Asc/Casp1/11*^*-/-*^ macrophages were as susceptible as *Nlrc4*^*-/-*^, whereas *Casp1/11*^*-/-*^ cells were partially restrictive (Fig 5C and 5D). Similar experiments performed with *Aim2/Casp1/11*^*-/-*^ macrophages did not support the role of AIM2 in the NLRC4/ASC-dependent growth restriction that occurred in the absence of caspase-1/11 (S6 Fig). These data indicate that flagellated species of *Legionellae* trigger NLRC4 responses that are independent of caspase-1/11 but dependent on ASC. To further confirm the participation of caspase-8 in this NLRC4/ASC inflammasome, we silenced caspase-8 in *Casp1/11*^*-/-*^ and *Asc/Casp1/11*^*-/-*^ macrophages. We confirmed the silencing by western blot analysis (Fig 5E and S7 Fig) and found that the reduction in caspase-8 expression impaired the restriction of bacterial replication in *Casp1/11*^*-/-*^ infected with *fliI*^*-*^ (Fig 5F). Inhibition of caspase-8 expression affected neither the replication of *fliI*^*-*^ in *Asc/Casp1/11*^*-/-*^ cells (Fig 5G) nor the replication of *flaA*^*-*^ in *Casp1/11*^*-/-*^ and in *Asc/Casp1/11*^*-/-*^ cells (Fig 5H and 5I). Collectively, these data are consistent with the hypothesis that flagellin activates a response that is dependent on NLRC4, ASC and caspase-8 and occurs in the absence of caspase-1/11.

**Fig 6:**
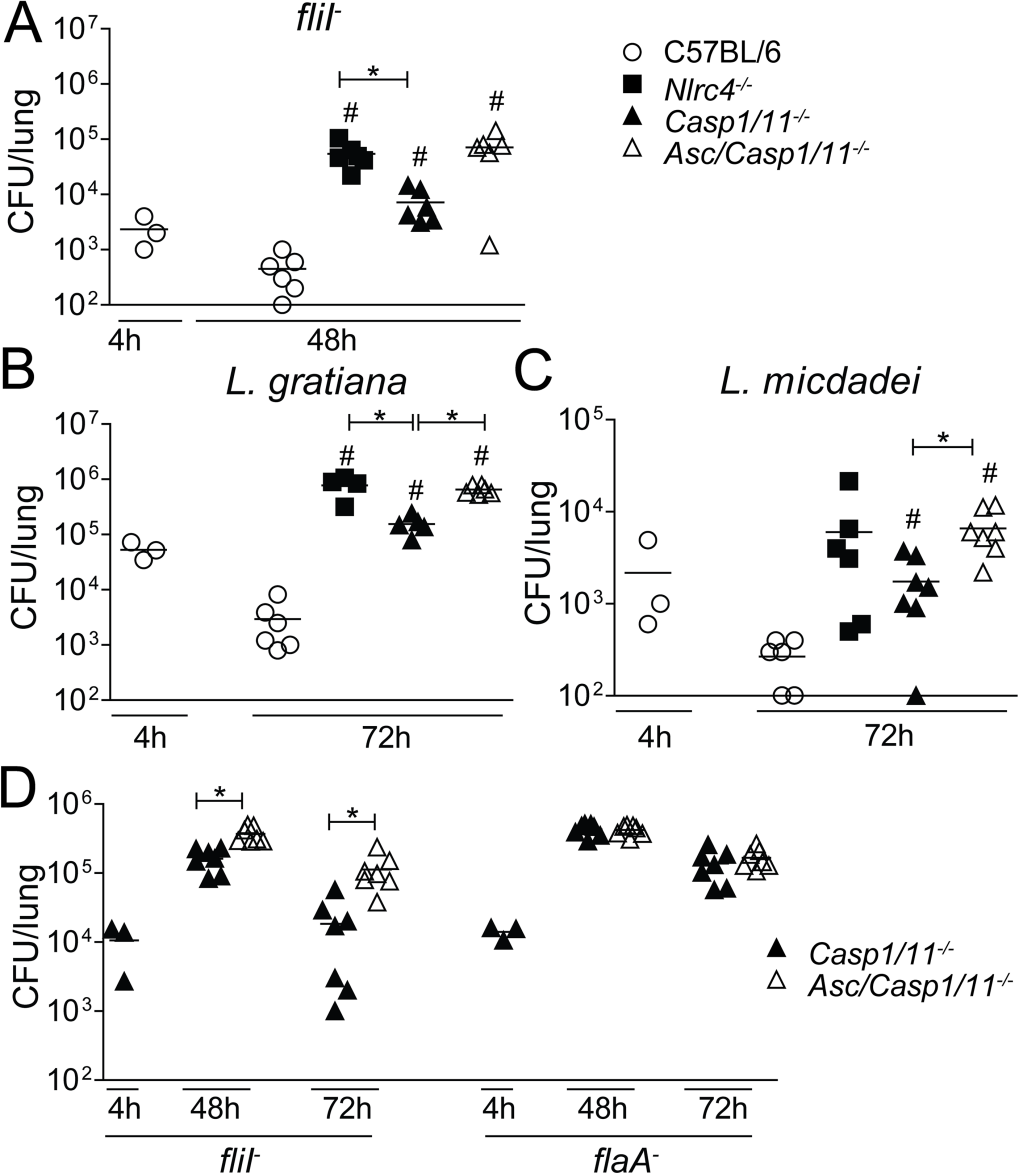
ASC is essential for NLRC4-mediated restriction *of L*. *pneumophila* replication independently of caspase-1/11 in vivo. C57BL/6 (open circles), *Nlrc4*^-/-^ (closed squares), *Casp1/11*^-/-^ (closed triangles) and *Asc/Casp1/11*^-/-^ (open triangles) mice were infected with motility-deficient *L*. *pneumophila* mutants expressing flagellin (*fliI*^-^, A, D), with flagellin-deficient *L*. *pneumophila* (*flaA*^*-*^, D), with *L*. *gratiana* (B) or with *L*. *micdadei* (C) at a dose of 10^5^ bacteria/mice. The mice were euthanized at 4, 48 or 72 hours after infection. Dilutions of the lung homogenates were added to charcoal-yeast extract agar plates for colony-forming unit determination. Each dot represents a single animal, and the horizontal lines represent the averages. *, *P*<0.05. ^#^, indicates *P*<0.05 compared with C57BL/6, Mann Whitney test. Data are presented for one representative experiment of five (A), one (B-C) and two (D) experiments performed with similar results.

*Casp8*^*-/-*^ mice are embryonic lethal [59], and we were not able to generate *Casp8/1/11*^*-/-*^ mice. Since the deletion of ASC impairs the assembly of the NLRC4/ASC/caspase-8 inflammasome and caspase-8 activation, we used *Asc/Casp1/11*^*-/-*^ mice to assess the role of the NLRC4/ASC/caspase-8 inflammasome in the restriction of *Legionella* replication in vivo. Using flagellin-positive bacteria such as *L*. *pneumophila*, *L*. *gratiana* and *L*. *micdadei*, we demonstrated that *Asc/Casp1/11*^*-/-*^ mice were highly permissive to bacterial replication and phenocopied infection of *Nlrc4*^*-/-*^ mice (Fig 6A–6C). *Casp1/11*^*-/-*^ mice were more permissive than C57BL/6, but they were less permissive than *Nlrc4*^*-/-*^ mice (Fig 6A–6C). Experiments performed with *fliI*^-^ and *flaA*^*-*^ indicated that *Asc/Casp1/11*^*-/-*^ were more permissive than *Casp1/11*^*-/-*^ when infected with *fliI*^-^ but not *flaA*^*-*^ (Fig 6D). To determine whether AIM2 accounted for the restriction of bacterial replication in the absence of caspase-1/11, we compared infection of *Aim2/Casp1/11*^*-/-*^ with *Casp1/11*^*-/-*^. We found that *Aim2/Casp1/11*^*-/-*^ and *Casp1/11*^*-/-*^ supported similar replication levels of *fliI*^*-*^ *L*. *pneumophila* in the lungs. In contrast, *Asc/Casp1/11*^*-/-*^ and *Nlrc4*^*-/-*^ mice were significantly more permissive to bacterial replication (S8 Fig). Collectively, these data indicate that AIM2 is dispensable for the functions of the NLRC4/ASC/caspase-8 inflammasome. This molecular platform is assembled in response to flagellin-positive bacteria and operates to restrict bacterial replication in vitro and in vivo in a process that is independent of both caspase-1 and caspase-11.

### Activation of the NLRC4/ASC/caspase-8 inflammasome triggers pore formation and cell death

**Fig 7:**
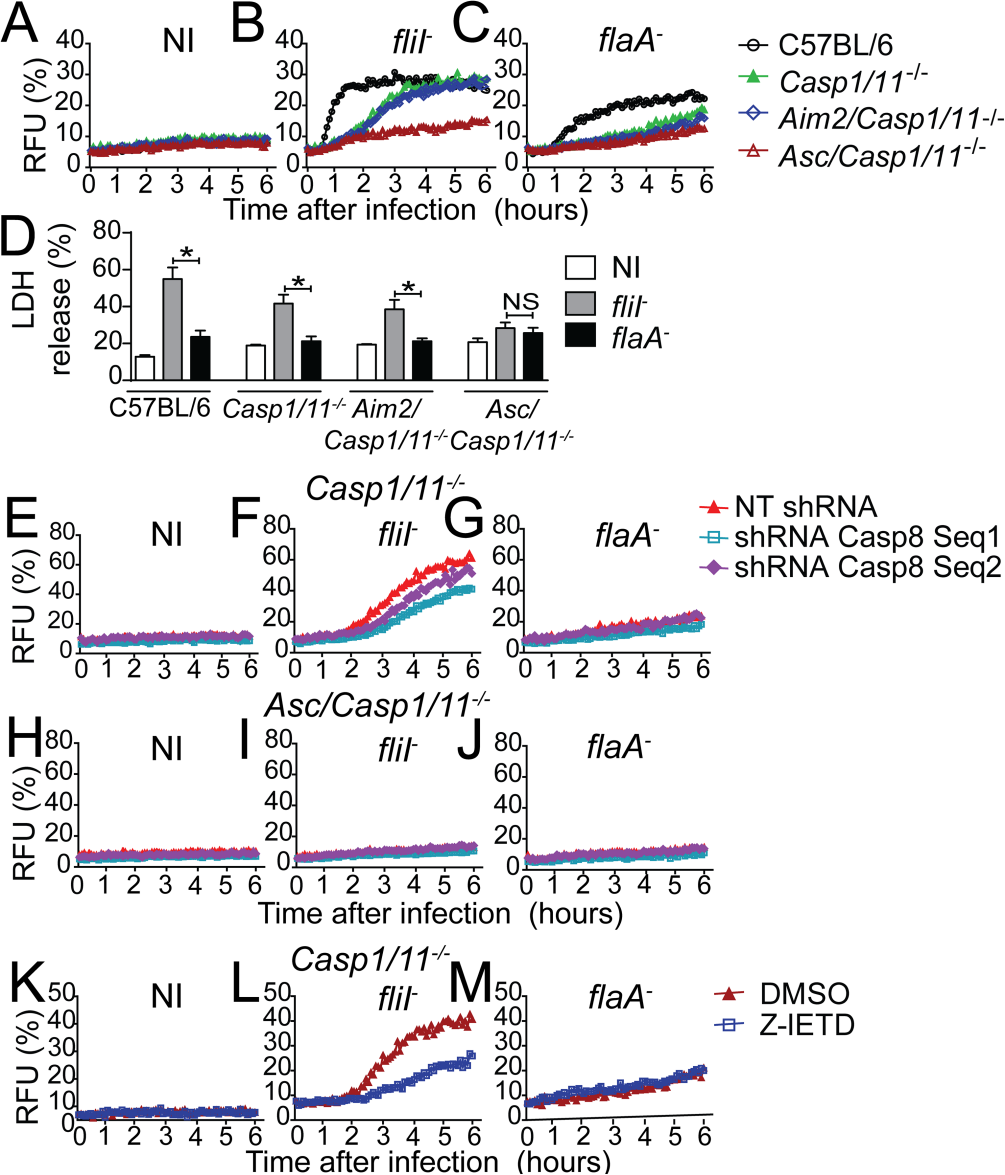
The NLRC4/ASC/caspase-8 inflammasome is important for pore formation and cell death independently of caspase-1/11. (A-D; K-M) Bone marrow-derived macrophages (BMDMs) were generated from C57BL/6, *Casp1/11*^*-/-*^, *Aim2/Casp1/11*^*-/-*^ and *Asc/Casp1/11*^*-/-*^ mice and infected with motility-deficient *L*. *pneumophila* mutants expressing flagellin (*fliI*^-^) or with flagellin-deficient bacteria (*flaA*^*-*^) at a MOI of 5. (E-J) BMDMs were transduced with retrovirus encoding shRNA sequences to target caspase-8 (Seq1, Seq2) and a non-target shRNA sequence (NT) and infected with *fliI*^-^ or *flaA*^*-*^ at a MOI of 5. (A-C; E-J) Pore formation was assessed fluorometrically in real time by the uptake of propidium iodide. RFU (%) represents the percentage of RFU estimated with cells lysed with Triton X-100. (D) LDH release was measured using the CytoTox 96 LDH-release kit. The LDH release (%) represents the percentage of LDH release estimated with cells lysed with Triton X-100. (K-M) BMDMs were treated with 50 μM of Z-IETD or DMSO for 1 hour and infected with (L) *fliI*^-^ or (M) *flaA*^*-*^ at a MOI of 5. Pore formation was assessed fluorometrically in real time by the uptake of propidium iodide. Data show the average ± SD of triplicate wells. *, *P*<0.05, Student´s t test. NS, not significant; RFUs, relative fluorescence units; NI, uninfected. Data are presented for one representative experiment of five (A-C), three (E-J) and two (D and K-M) experiments with similar results.

Activation of caspase-1 inflammasomes induces pyroptosis and contributes to the restriction of infection by flagellated bacteria such as *L*. *pneumophila*, *Salmonella typhimurium* and *Burkholderia thailandensis* [23]. Accordingly, we have previously demonstrated that *L*. *pneumophila* trigger pyroptosis in a process mediated by caspase-1 and caspase-11, which are activated in response to flagellin and LPS, respectively [20,24,35,61]. Thus, we investigated whether activation of the NLRC4/ASC/caspase-8 inflammasome could trigger cell death in *Casp1/11*^*-/-*^ macrophages. We assessed membrane permeabilization fluorometrically in real time via the uptake of propidium iodide. Macrophages were infected with *fliI*^*-*^ or *flaA*^*-*^, and pore formation was monitored in real time for 6 hours. C57BL/6 macrophages triggered robust pore formation in response to infection with *fliI*^*-*^ and reduced pore formation in response to *flaA*^*-*^ (Fig 7A–7C). The pore formation observed in C57BL/6 macrophages infected with *flaA*^-^ mutants was dependent on caspase-11 [61] and will not be addressed herein. Importantly, despite the absence of both caspase-1 and caspase-11, we detected significant pore formation in *Casp1/11*^*-/-*^ cells infected with *fliI*^*-*^ (Fig 7B). This response was not detected in *Casp1/11*^*-/-*^ cells infected with *flaA*^*-*^ or in *Asc/Casp1/11*^*-/-*^ macrophages infected with *fliI*^*-*^ or *flaA*^*-*^ (Fig 7B and 7C). These data support the hypothesis that NLRC4/ASC/caspase-8 induces pore formation. Experiments performed using *Aim2/Casp1/11*^*-/-*^ macrophages corroborate our previous findings, indicating that AIM2 is not required for the activities of the NLRC4/ASC/caspase-8 inflammasome (Fig 7B and 7C). Pore formation induced in response to caspase-1 and caspase-11 activation culminates with the induction of macrophage lysis, a process that can be assessed by the presence of LDH in tissue culture supernatants [24,61,62]. Thus, we measured LDH release in the supernatants of macrophages infected with *fliI*^-^ and *flaA*^-^ for 8 hours. By comparing *Casp1/11*^*-/-*^ and *Asc/Casp1/11*^*-/-*^ cells, we found that macrophage lysis occurred despite the absence of caspase-1/11 (Fig 7D). Cell death was flagellin-dependent because infections with *fliI*^-^ but not *flaA*^-^ induced LDH release (Fig 7D). The participation of caspase-8 in pore formation and cell death induced in caspase-1/11-deficient macrophages was evident using both MOI 5 (Fig 7) and MOI 10 (S9 Fig). Importantly, cell death was not observed in *Asc/Casp1/11*^*-/-*^, a feature that corroborates the pore formation studies and indicates that the NLRC4/ASC/caspase-8 inflammasome triggers pore formation and lysis of infected cells. We also assessed whether the NLRC4/ASC/caspase-8 inflammasome was important for the activation of inflammatory cytokines. We found that whereas C57BL/6 macrophages readily triggered the production of IL-1β after 24 hours of infection with flagellated bacteria, the *Casp1/11*^*-/-*^ or *Asc/Casp1/11*^*-/—*^deficient cells do not trigger a IL-1β production (S10A Fig). The production of IL-12p40 by these cells confirmed that all macrophages were primed and could respond to *L*. *pneumophila* infection (S10B Fig). To evaluate the participation of caspase-8 in cell death induced by the NLRC4/ASC/caspase-8 inflammasome, we silenced endogenous caspase-8 using shRNA. Macrophages that were transduced with retrovirus encoding shRNA did not exhibit pore formation before infection, indicating that transduction itself did not trigger cell death (Fig 7E and 7H). In contrast, pore formation was evident in *Casp1/11*^*-/-*^ but not in *Asc/Casp1/11*^*-/-*^ macrophages in response to *fliI*^-^ infection (Fig 7F and 7I). Pore formation induced in *Casp1/11*^*-/-*^ was diminished in caspase-8-silenced cells (Fig 7F). In support of the role of flagellin for triggering these responses, we did not detect pore formation in cells infected with *flaA*^-^ (Fig 7G and 7J). To further evaluate the participation of caspase-8 in pore formation induced by flagellin, we performed pore formation assays using Z-IETD, a cell permeable peptide that binds irreversibly to the catalytic site of caspase-8 [63–65]. We found that treatment of *Casp1/11*^*-/-*^ macrophages with DMSO or Z-IETD did not cause pore formation in uninfected cells (Fig 7K). However, Z-IETD treatment reduced the pore formation induced by *fliI*^*-*^ (Fig 7L) but not by *flaA*^*-*^ (Fig 7M). Collectively, these data indicate that flagellin-positive bacteria trigger pore formation and cell death-independent of caspase-1/11 via a process that requires ASC and caspase-8.

### Naip5 is required for NLRC4/ASC/caspase-8 inflammasome activation in response to flagellated Legionella

**Fig 8:**
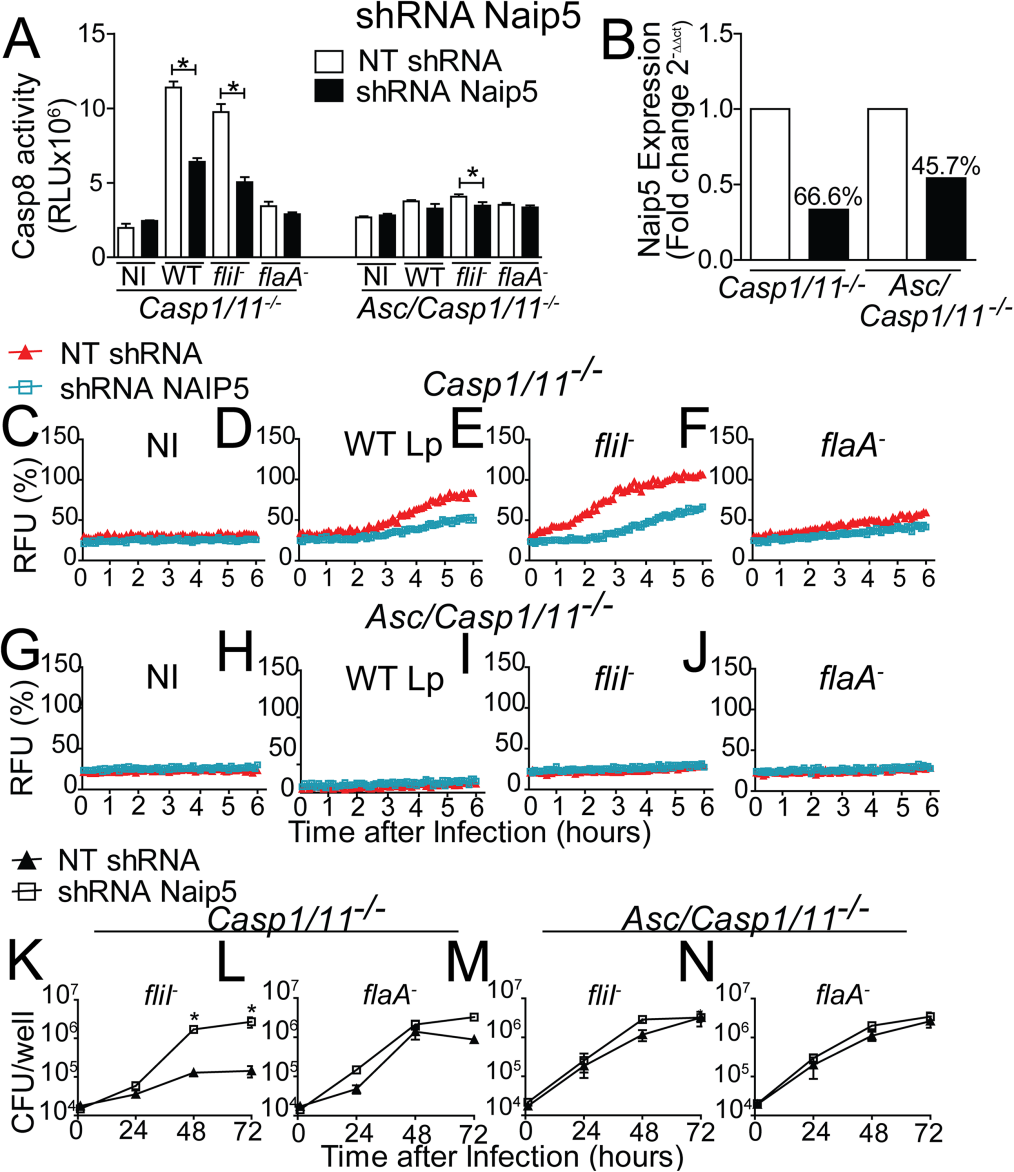
Naip5 is required for the functions of the NLRC4/ASC/Caspase-8 inflammasome independently of caspase-1/11. Bone marrow-derived macrophages (BMDMs) generated from *Casp1/11*^*-/-*^ and *Asc/Casp1/11*^*-/-*^ mice were transduced with a retrovirus encoding shRNA sequence to target Naip5 and a non-target shRNA sequence (NT). Transduced BMDMs were infected with wild-type *L*. *pneumophila* (WT Lp), motility-deficient mutants expressing flagellin (*fliI*^-^) or with flagellin-deficient mutants (*flaA*^*-*^) at a MOI of 10. (A) 8 hours after infection the activity of caspase-8 was measured using the Caspase-8 Glo Assay. (B) Quantification of the *Naip5* gene expression by Real Time qPCR. *Gapdh* gene was used as a control for normalization of expression levels. The number above the bars indicates the percentage of silencing compared to the NT shRNA (open bars). (C-J) Pore formation was assessed fluorometrically in real time by the uptake of propidium iodide. The RFU (%) represents the percentage of RFU estimated with cells lysed with Triton X-100. (K-N) The cells were infected with *fliI*^-^ or *flaA*^-^ for 24, 48 and 72 hours for CFU determination. Data show the average ± SD of triplicate wells. *, *P*<0.05, Student´s t test (A) and ANOVA (K-N). NS, not significant; RFU, relative fluorescence units; NI, uninfected. Data are presented for one representative experiment of two (A-J) and three (K-N) experiments with similar results.

Our data reveal that the NLRC4/ASC/caspase-8 inflammasome is activated in *Casp1/11*^*-/-*^ macrophages in response to infection with flagellated *Legionella*. To evaluate if Naip5 is required for activation of this inflammasome, we used shRNA to silence endogenous Naip5. In Naip5 silenced *Casp1/11*^*-/-*^ macrophages, we detected a reduced activation of caspase-8 in response to WT Lp and *fliI*^*-*^ bacteria (Fig 8A). Naip5 silencing was confirmed by RT-PCR (Fig 8B). We also tested if Naip5 is important for pore formation induced via caspase-8 in *Casp1/11*^*-/-*^ macrophages. By evaluating pore formation, we found that Naip5 is important for efficient pore formation in response to WT Lp and *fliI*^*-*^ bacteria (Fig 8D–8E). As previously reported, no pore formation was detected in response to *flaA*^*-*^ bacteria (Fig 8F) or in Asc/*Casp1/11*^*-/-*^ macrophages (Fig 8G–8J). Finally, we tested if Naip5 is important for restriction of *L*. *pneumophila* replication in *Casp1/11*^*-/-*^ macrophages. We found that Naip5 is important for restriction of flagellin-positive *L*. *pneumophila* replication in *Casp1/11*^*-/-*^ macrophages (Fig 8K). Naip5 silencing did not affect the replication of *flaA*^*-*^ bacteria in *Casp1/11*^*-/-*^ macrophages (Fig 8L). As predicted, Asc/*Casp1/11*^*-/-*^ macrophages were permissive to replication of both *flaA*^*-*^ and *fliI*^*-*^ bacteria and Naip5 did not influenced this process (Fig 8M and 8N). Taken together, these data indicates that Naip5 participate of the NLRC4/ASC inflammasome that trigger caspase-8 activation in the absence of caspase-1/11.

### Caspase-8 is recruited to the NLRC4/ASC/caspase-1 inflammasome but it is only activated when caspase-1 or gasdermin-D is suppressed

**Fig 9:**
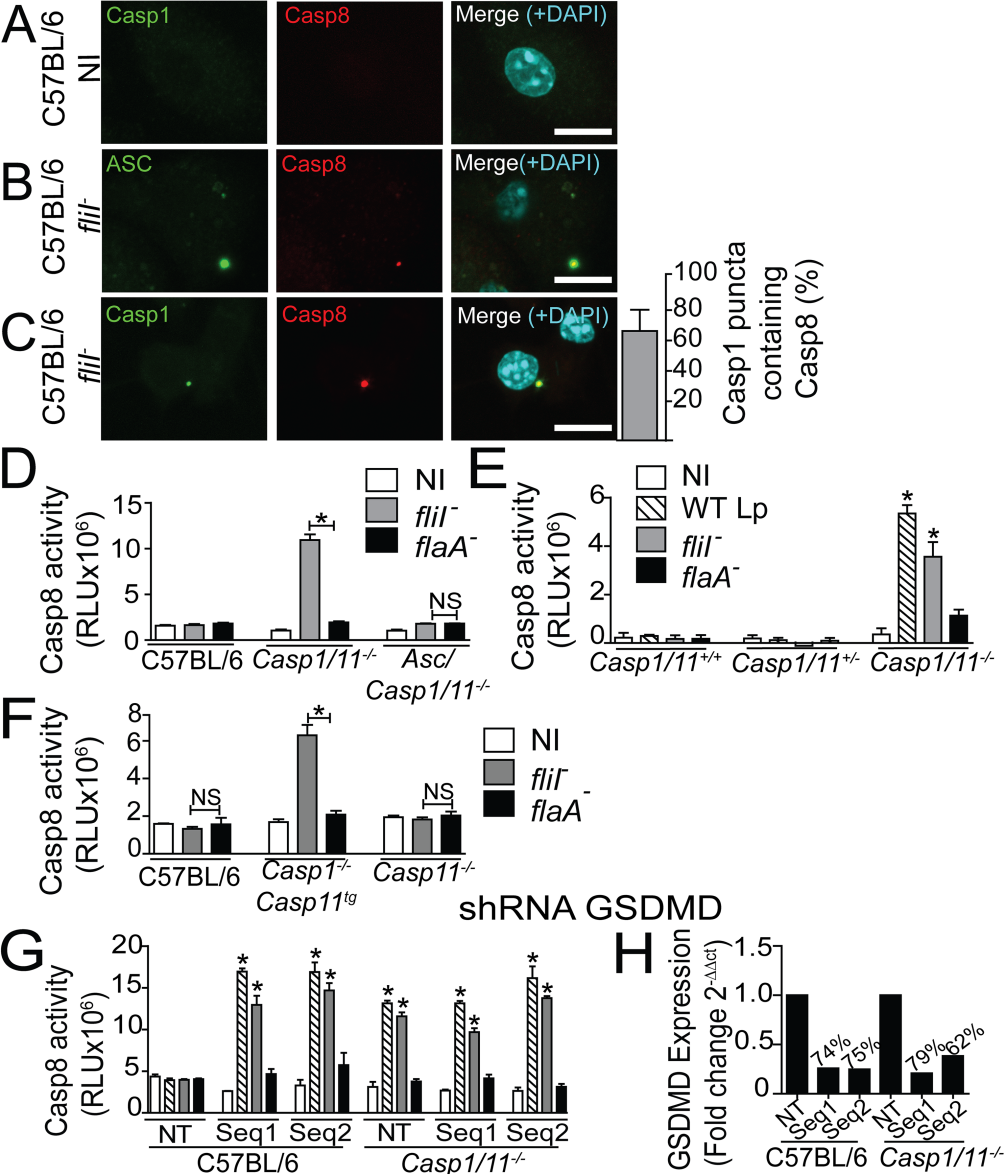
Caspase-8 colocalizes with the NLRC4/ASC/Caspase-1 inflammasome, but it is only activated if caspase-1 or gasdermin-D is inhibited. (A-C) Bone marrow-derived macrophages (BMDMs) generated from C57BL/6 mice were infected with motility-deficient *L*. *pneumophila* mutants expressing flagellin (*fliI*^-^) at a MOI of 10 for 8 h. The cultures were stained with anti-caspase-1 (green) (A, C) or anti-ASC (green) (B), anti-caspase-8 (red). Cell nuclei were stained with DAPI (cyan). Images were acquired by multiphoton microscopy with a 63x oil immersion objective and analyzed using ImageJ software. The images are the maximal projection of a z project. Scale bars, 10μm. (C) The percentage of the caspase-1 puncta containing caspase-8 was determined using an epifluorescence microscope. (D) BMDMs were generated from C57BL/6, *Casp1/11*^*-/-*^ and *Asc/Casp1/11*^*-/-*^ mice and infected with *fliI*^-^ or *flaA*^*-*^. (E) BMDMs were generated from *Casp1/11*^*+/+*^, *Casp1/11*^*+/-*^ and *Casp1/11*^*-/-*^ littermate control mice and infected with WT, *fliI*^*-*^ and *flaA*^*-*^. (F) BMDMs were generated from C57BL/6, *Casp1*^*-/-*^*Casp11*^*tg*^ and *Casp11*^*-/-*^ mice and infected with WT, *fliI*^*-*^ and *flaA*^*-*^. (G-H) BMDMs generated from C57BL/6 and *Casp1/11*^*-/-*^ mice were transduced with a retrovirus encoding shRNA sequences to target Gasdermin D (GSDMD) (Seq1, Seq2) and a non-target shRNA sequence (NT). (G) Transduced cells were infected with WT Lp, *fliI*^*-*^ and *flaA*^*-*^. (D-G) Cells were infected with an MOI of 10 and after 8 hours the activity of caspase-8 was measured using the Caspase-8 Glo Assay. (H) GSDMD silencing was confirmed by Real Time qPCR. *Gapdh* gene was used as a control for normalization of expression levels. The numbers above the bars indicate the percentage of silencing compared to the NT sequence. Data show the average ± SD of triplicate wells. *, *P*<0.05, Student´s t test in relation to *flaA*^*-*^. RLU, relative luminescence units; NI, non-infected. Data are presented for one representative experiment of two (A-C and E-H) and five (D) experiments with similar results.

In the experiments shown thus far, we used *Casp1/11*^*-/-*^ macrophage as a tool to assess the caspase-8 effects without the interference of caspase-1. However, because caspase-1 is present in natural conditions, we tested if caspase-8 participates in the NLRC4/ASC inflammasome in the presence of caspase-1. First, we infected C57BL/6 macrophages with flagellin-positive *L*. *pneumophila* to assess if endogenous caspase-8 colocalizes with the Naip5/NLRC4/ASC inflammasome. In uninfected conditions, we detected no significant puncta formation (Fig 9A). However, in response to *fliI*^*-*^ bacteria, caspase-8 colocalizes with ASC (Fig 9B) and caspase-1 (Fig 9C). We determined that caspase-8 is present in more than 60% puncta containing caspase-1 (Fig 9C). These data indicates that regardless to the presence of caspase-1, the caspase-8 is recruited to the inflammasome during activation.

Next, we evaluated caspase-8 activation in wild-type macrophages. We found that caspase-8 activation does not occur in C57BL/6 macrophages infected with *L*. *pneumophila*. As expected caspase-8 is readily activated in *Casp1/11*^*-/-*^, but not in *Asc*/*Casp1/11*^*-/-*^ macrophages infected with *fliI*^*-*^ bacteria (Fig 9D). To ensure similar genetic background, we intercrossed a F1 progeny of *Casp1/11*^*-/-*^ x C57BL/6 to obtain F2 littermate controls. Infections in macrophages from littermate control mice indicated that caspase-8 activation occur in *Casp1/11*^*-/-*^, but not in *Casp1/11*^*+/-*^ and *Casp1/11*^*+/+*^ macrophages (Fig 9E). To further test whether the deficiency in caspase-1 or caspase-11 enable caspase-8 activation, we performed experiments using mice single deficient in caspase-1 or caspase-11 as previously described [35,66]. We found that caspase-11 deficiency alone is not sufficient to enable caspase-8 activation in response to *L*. *pneumophila* infection (Fig 9F). In contrast, caspase-1-deficient cells expressing caspase-11 as a transgene (*Casp1*^*-/-*^*Casp11*^*tg*^) effectively trigger caspase-8 activation in response to *fliI*^*-*^ *L*. *pneumophila* (Fig 9F). These data indicate that caspase-1 but not caspase-11 is required to prevent caspase-8 activation.

Caspase-1 activation via the NLRC4 inflammasome is known to trigger activation of gasdermin-D (GSDMD) to induce cell death [67,68]. Thus, we tested if inhibition of GSDMD is sufficient to enable caspase-8 activation in the presence of caspase-1. To achieve this, we inhibited endogenous GSDMD using shRNA and found that despite the presence of caspase-1, caspase-8 is robustly activated when GSDMD is inhibited (Fig 9G). RT-PCR was used to confirm the silencing of the two different sequences of shRNA used (Fig 9H). To further confirm the GSDMD silencing, we performed pore formation assay in C57BL/6 macrophages infected with *L*. *pneumophila*. We found that GSDMD silencing inhibited the caspase-1-mediated pore formation induced by WT Lp and *fliI*^*-*^ *L*. *pneumophila* (S11A–S11D Fig). However, GSDMD did not participate of pore formation induced via caspase-8 that occurs in *Casp1/11*^*-/-*^ macrophages (S11E–S11H Fig). Collectively, these data indicates that despite the presence of caspase-1, caspase-8 activation occur in the Naip5/NLRC4/ASC inflammasome when GSDMD is inhibited.

## Discussion

The recognition of *Legionella* flagellin by the Naip5/NLRC4 inflammasome in macrophages is a major mechanism for the restriction of bacterial replication in mouse cells [17–20,22,69,70]. It is well accepted in the field that not all NLRC4 functions require caspase-1 [29,38,71,72]. This conclusion is supported by the observation that *Nlrc4*^*-/-*^ mice (and their macrophages) are significantly more permissive to *L*. *pneumophila* replication than *Casp1/11*^*-/-*^ mice [29,38]. Here, we unraveled this caspase-1/11-independent pathway and characterized an inflammasome composed of Naip5, NLRC4, ASC and caspase-8, which operates in the absence of caspase-1 and caspase-11. This inflammasome effectively participates in the mechanisms involved in the restriction of bacterial replication in macrophages and in vivo. Previous biochemistry studies using yeast two-hybrid screening showed that NLRC4 is ubiquitinated by Sug1, a process that facilitates the activation of caspase-8 [51]. Thus, it is possible that Sug1 is also a component of this NLRC4 inflammasome. In addition, previous studies using the *Salmonella enterica* serovar Typhimurium have indicated that both caspase-8 and caspase-1 are recruited to the ASC puncta in response to infection. However, caspase-8 is involved in the synthesis of pro-IL-1β and is dispensable for *Salmonella*-induced cell death [32]. These data contrast with published data using *L*. *pneumophila*, which indicate that in the absence of caspase-1/11, no inflammatory cytokines are produced [20,21,25,30,35,66]. Accordingly, our data indicates that this Naip5/NLRC4/ASC/Caspase-8 inflammasome is very inefficient to trigger IL-1β maturation when caspase-1 is not present.

Importantly, AIM2 is not part of this NLRC4/ASC/caspase-8 inflammasome. AIM2 is well-known to trigger caspase-8 activation via ASC [33,34,58]. Our data unequivocally demonstrate that AIM2 is neither a component of this inflammasome, nor is it required for inflammasome functions. AIM2 did not colocalize with the NLRC4/ASC/caspase-8 puncta, it was dispensable for the activation of caspase-8 in response to flagellated *L*. *pneumophila* and for the induction of cell death and restriction of *L*. *pneumophila* replication. The pyrin domain of ASC can bind to the death domain of caspase-8 [46]. Thus, it is possible that the interactions of the ASC pyrin domain with the caspase-8 dead domain are critical for the recruitment of caspase-8 to the complex. Consistent with this hypothesis, our studies unequivocally show that ASC is required for the assembly and function of this NLRC4 inflammasome. Importantly, the characterization of this NLRC4/ASC/caspase-8 inflammasome accounted to clarify a controversy in the field concerning the participation of ASC in the NLRC4 inflammasome. Studies utilizing biochemistry and cells from gene-deficient mice have demonstrated that ASC is dispensable for NLRC4 functions, including pyroptosis and the restriction of *L*. *pneumophila* replication [20,21,29]. However, ASC is essential for caspase-1 cleavage and the processing of inflammatory cytokines in response to flagellated *L*. *pneumophila*, a process that is NLRC4-dependent and NLRP3-independent [20,21,25,30]. In addition, ASC participates in the restriction of intracellular replication of *L*. *pneumophila* under certain circumstances [36,37]. Our studies provide data that help to consolidate these data in a cohesive model. NLRC4 can operate to form an inflammasome in absence of ASC that triggers pore formation and the restriction of bacterial replication [20,21,25,30]. This platform does not form puncta and is ineffective for triggering caspase-1 cleavage and processing inflammatory cytokines. When ASC is present, NLRC4 inflammasome associates with ASC and recruit caspase-1 and caspase-8 to the puncta. This inflammasome is very efficient to cleave caspase-1 and inflammatory cytokines such as IL-1β. Interestingly, our data indicate that caspase-8 is not activated when caspase-1 is present. However, when caspase-1 is missing or when Gasdermin-D is inhibited, we detected a robust caspase-8 activation. These data suggest that activation of caspase-8 in the Naip5/NLRC4/ASC inflammasome functions as a backup strategy to guarantee cell death when key components of the pyroptotic cell death are inhibited. Interestingly, our data and previously published data indicate that gasdermin-D is dispensable for caspase-8-induced cell death [73]. Therefore, when gasdermin-D is inhibited, caspase-8 engages gasdermin-D-independent cell death. It is possible that caspase-8 induces caspase-3 and caspase-7 to targed gasdermin-E (also known as DFNA5) to induce pore formation and cell death independent of gasdermin-D [74,75]. This may guarantee appropriate responses to pathogens that inhibit canonical components of pyroptotic cell death such as caspase-1 or gasdermin-D.

## Materials and methods

### Bacterial culture

The *L*. *pneumophila* bacteria used were JR32 and isogenic clean deletion mutants for motility (*fliI*^-^) and flagellin (*flaA*^*-*^) [19,38]. *L*. *micdadei* (ATCC 33218) and *L*. *gratiana* (ATCC 49413) were used to generate streptomycin-resistant strains. *RpsL* mutants of *L*. *micdadei* and *L*. *gratiana* were isolated by plating these strains on CYE agar containing 100 μg/ml of streptomycin. All bacteria were grown on buffered charcoal-yeast extract (CYE) agar plates [1% yeast extract, 1% MOPS, 3.3 mM L-cysteine, 0.33 mM Fe(NO3)3, 1.5% Bacto agar and 0.2% activated charcoal, pH 6.9] [76].

### Macrophages

Bone Marrow derived macrophages (BMDMs) were generated from mice as previously described [77]. Briefly, bone marrow cells were harvested from femurs and differentiated with RPMI 1640 (Gibco) containing 20% fetal bovine serum (FBS—Invitrogen) and 30% L-929 cell-conditioned medium (LCCM), 2 mM L-glutamine (Sigma-Aldrich), 15 mM Hepes (Gibco) and 100 U/ml penicillin-streptomycin (Sigma-Aldrich) at 37°C with 5% CO_2_ [77]. BMDMs were seeded at 2 X 10^5^ cells/well in 24-well plates and cultivated in RPMI 1640 medium (Gibco) supplemented with 10% FBS, 5% LCCM, 2 mM L-glutamine and 15 mM Hepes.

### In vitro infections and CFU determination

For the in vitro infections, the cultures were infected at a multiplicity of infection of 0.015, 5 or 10 followed by centrifugation for 5 minutes at 300 X *g* at room temperature and incubation at 37°C in a 5% CO_2_ atmosphere. In the colony-forming units (CFU) experiments, cultures infected at a MOI of 10 were washed two times with PBS, and 1 ml of medium was added to each well. For CFU determination, the cultures were lysed in sterile water, and the cell lysates were combined with the cell culture supernatant from the respective wells. Lysates plus supernatants from each well were diluted in water, plated on CYE agar plates, and incubated for 4 days at 37°C for CFU determination as described previously [28,38].

### Retroviral transduction and quantification of NLRC4-GFP and ASC-GFP puncta

Murine *Nlrc4* or *Asc* were cloned into the pEGFP (N2) vector (Clontech) using XhoI and BamHI restriction sites as previously described [30]. NLRC4-GFP or ASC-GFP and GFP were cloned into the pMSCV2.2 murine-specific retroviral vector (Clontech). The pCL vector system 51 was used to package the retroviruses in transfected monolayers of Hek Peak cells (ATCC CRL-2828), which were maintained in RPMI with 10% FBS. The supernatant from the Hek Peak cells containing retrovirus was collected three days after transfection, filtered using a 0.45-μm filter and used for BMDM transduction. BMDMs were obtained from *Casp1/11*^-/-^, *Asc/Casp1/11*^-/-^ and *Aim2/Casp1/11*^-/-^ mice and seeded in differentiation medium. On day 3 of differentiation, the supernatants containing retroviral were added to BMDMs in 20% FBS and 25% LCCM. After differentiation, the BMDMs were seeded at 2 X 10^5^ cells/well in 24-well plates containing 12-mm glass cover slides and cultivated in RPMI 1640 medium supplemented with 10% FBS and 5% LCCM. For the caspase colocalization experiments, the cultures were treated with 20 μm of Z-VAD for 1 hour and infected at a MOI of 1, 3 or 10. After infection, the plates were centrifuged for 5 minutes at 300 X *g* at room temperature and incubated at 37°C in a 5% CO_2_ atmosphere. At 1, 2, 4 and 8 hours after infection, the cells were fixed with 4% paraformaldehyde, permeabilized with 0.05% saponin, stained with DAPI and mounted on glass slides using Prolong Gold Antifade Reagent (Invitrogen). For the colocalization assay, the cells were stained with rat anti-caspase-8 (Enzo– 1G12; 1:50), rabbit anti-cleaved caspase-8 (Cell signaling- 8592; 1:800), rabbit anti-cleaved caspase-3 (Cell signaling- 9664; 1:400); rabbit anti-cleaved caspase-7 (Cell signaling-8438; 1:400), anti-ASC (Adipogen- AL177; 1:250), goat anti-ASC (Santa Cruz–sc33958; 1:50), rabbit anti-caspase-1 (Santa Cruz- sc514; 1:500) or anti-AIM2 (Cell signaling- 13095; 1:400), followed by Alexa 594-conjugated goat anti-rabbit secondary Ab (Invitrogen; 1:3000), Alexa 594-conjugated goat anti-rat secondary Ab (Invitrogen; 1:3000) or Alexa 647-conjugated chicken anti-rabbit secondary Ab (Invitrogen; 1:2000) and DAPI and mounted on glass slides using Prolong Gold Antifade Reagent (Invitrogen). The images were processed using LAS AF software (Leica Microsystems) and analyzed under fluorescence using a Leica DMI 4000B inverted microscope with a 100X oil objective. The number of NLRC4-GFP or ASC-GFP puncta in the transduced cells and the colocalization were quantified. Bacteria were not stained. Therefore, the whole cell population was scored. Multiphoton microscopy images were acquired using an LSM 780 Zeiss AxioObserver microscope equipped with a 63X oil immersion objective and analyzed using ImageJ software.

### Retroviral silencing of caspase-8, Naip5 and GSDMD

For retroviral silencing of caspase-8, Naip5 and GSDMD (Gasdermin D), Hek Peak cells were transfected with lentiviral vectors encoding a small hairpin RNA (shRNA) targeting caspase-8 [Sigma- Seq1: TRCN0000231279 (Sequence- CCGGTCATCTCACAAGAACTATATTCTCGAGAATATAGTTCTTGTGAGATGATTTTTG); Seq2: TRCN0000231281 (Sequence–CCGGTCCTGACTGGCGTGAACTATGCTCGAGCATAGTTCACGCCAGTCAGGATTTTTG)], Naip5 [Sigma- TRCN0000114742 (Sequence—CCGGCGCTTGATTATCTTCTGGAAACTCGAGTTTCCAGAAGATAATCAAGCGTTTTTG)], GSDMD [Sigma- TRCN0000219619 (Sequence—CCGGGATTGATGAGGAGGAATTAATCTCGAGATTAATTCCTCCTCATCAATCTTTTTG); TRCN0000219620 (Sequence—CCGGCCTAAGGCTGCAGGTAGAATCCTCGAGGATTCTACCTGCAGCCTTAGGTTTTTG)] and a negative control vector that included a non-target shRNA sequence (NT). The plates were treated with polyethylenimine (Sigma-Aldrich) (Corning). Transduced cells were maintained in RPMI 1640 medium supplemented with 10% FBS at 37°C and 5% CO_2_. Lentiviruses expressing shRNAs were collected, filtered using a 0.45-μm filter and added to the BMDMs. After selection with puromycin, the resistant cells were seeded at 2 X 10^5^ cells/well in 24-well plates and infected with *fliI*^-^ or *flaA*^-^ for CFU determination. The caspase-8 silencing efficiency was measured by immunoblotting: 1 X 10^6^ cells were lysed in RIPA buffer (10 mM Tris-HCl, pH 7.4, 1 mM EDTA, 150 mM NaCl, 1% Nonidet P-40, 1% deoxycholate, and 0.1% SDS) in the presence of a protease inhibitor cocktail (Roche). The lysates were suspended in 4X Laemmli buffer, boiled for 5 minutes, resolved by 15% SDS PAGE and transferred (Semidry Transfer Cell, Bio-Rad) to 0.22-μm nitrocellulose membranes (GE Healthcare). The membranes were blocked in Tris-buffered saline (TBS) with 0.01% Tween-20 and 5% non-fat dry milk for 1 hour. Rat anti-caspase-8 p55 (Enzo– 1G12; 1:500) and anti-rat peroxidase-conjugated antibody (KPL, 1:3000) were diluted in blocking buffer for the incubations (overnight for anti-caspase-8 and 1 hour for the secondary antibody). The ECL luminol reagent (GE Healthcare) was used for antibody detection. For evaluation of caspase-8 silencing by immunoblots, Image J software were used to estimate the ratio of caspase-8 p55 to α-actin.

### Caspase-8 activation

To assess caspase-8 activation, BMDMs were infected with *fliI*^*-*^ or *flaA*^*-*^ for 8 hours, and the activity of caspase-8 was measured using the Caspase-8 Glo Assay (Promega) according to manufacturer´s recommendations. To evaluate caspase-8 activation by western blot analysis, 1 X 10^7^ cells were infected at a MOI of 10 and lysed 8 hours after infection in RIPA buffer with protease inhibitor, as previously described. The lysates were suspended in 4X Laemmli buffer, boiled for 5 minutes, resolved by 15% SDS PAGE and transferred to 0.22-μm nitrocellulose membranes. The membranes were blocked in Tris-buffered saline (TBS) with 0.01% Tween-20 and 5% non-fat dry milk or for 1 hour. The mouse anti-caspase-8 (Enzo– 1G12) and anti-rabbit peroxidase-conjugated antibody (KPL; 1:3000) were diluted in blocking buffer for the incubations (overnight for anti-cleaved caspase-8 and 1 hour for the secondary antibody). ECL luminol reagent (GE Healthcare) was used for antibody detection.

### Real Time q-PCR

Total RNA was extracted from 2 X 10^6^ macrophages using total RNA isolation kit (illustra RNAspin, GE Healthcare, UK), according to manufacturer’s instructions. After extraction, an aliquot of 2 μl was used to determine the RNA concentration in NanoDrop (Thermo Fisher Scientific) and 1 μg of the extracted RNA was used for the cDNA conversion using the iScriptTM cDNA Synthesis kit (BIO-RAD) in a thermal cycler. The cDNA (10 ng) was used for the quantification of the Caspase 8 gene expression (TaqMan Assay: Casp 8—Mm01255716_m1) by real-time PCR using TaqMan Fast Advanced Master Mix, according to the manufacturer's instruction (Applied Biosystems). Actin beta (Actb) gene (TaqMan Assay: Actb-Mm00607939_s1) was used as a control for normalization of expression levels. The quantification of GSDMD and Naip5 were performed using 25 ng of cDNA and 10 μM of each primer, 1X SYBR Green (Applied Biosystems), and was normalized using the housekeeping gene glyceraldehyde-3-phosphate dehydrogenase (Gapdh). The specificity of the PCR products was assessed by melting curve analysis for all samples. The following primers were used: Gapdh, FWD- AGGTCGGTGTGAACGGATTTG, REV–TGTAGACCATGTAGTTGAGGTCA, GSDMD, FWD–TCATGTGTCAACCTGTCAATCAAGGACAT, REV- CATCGACGACATCAGAGACTTTGAAGGA, NAIP5, FWD- GTTGAGATTGGAGAAGACCTCG, REV-CACACGTGAAAGCAACCATGG. The real-time quantitative reaction was performed in the Viia 7 Real-Time PCR System (Applied Biosystems). The results were analyzed using the 2^-ΔΔCT^ method and are expressed in relation to the reference group. The percentage of silencing knockdown was estimated using the [1-(2^-ΔΔCT^) x 100] equation [78].

### Mice and in vivo infections

Mice used in this study were breed and maintained in institutional animal facilities. Mice used were C57BL/6 (Jax 000664), *Nlrc4*^-/-^ [79], *Casp1/11*^-/-^ [80], *Asc*^-/-^ [81], *Casp11*^*-/-*^ and *Casp1*^*-/-*^*Casp11*^*tg*^ [66] were. Double-deficient mice were generated by intercrossing a F1 progeny of the parental strains. All mice were matched by sex and age (all were at least 8 weeks old at the time of infection) and were in a C57BL/6 mouse genetic background. For the in vivo experiments, approximately 5–7 mice per group were used, as indicated in the figures. For in vivo infections, the mice were anesthetized with ketamine and xylazine (50 mg/kg and 10 mg/kg, respectively) by intraperitoneal injection followed by intranasal inoculation with 40 μl of phosphate-buffered saline (PBS) containing 1 X 10^5^ bacteria per mouse. For CFU determination, the lungs were harvested and homogenized in 5 ml of sterile water for 30 seconds using a tissue homogenizer (Power Gen 125; Thermo Scientific). Lung homogenates were diluted in sterile water and plated on CYE agar plates for CFU determination as previously described [28,38].

### Pore formation assay

Pore formation in BMDMs was quantified based on the permeability to propidium iodide (PI) in damaged cells as previously described [61]. BMDMs were seeded in a black, clear-bottom 96-well plate (1 X 10^5^ cells/well). Before infection, the medium was replaced with 10% RPMI without phenol red, 0.038 g/ml NaHCO_3_, 6 μl/ml PI and anti-*L*. *pneumophila* (1:1000). Infected BMDMs were maintained at 37 °C, and PI was excited at 538 nm. The fluorescence emission was read at 617 nm every 5 minutes using a plate fluorometer (SpectraMax i3x, Molecular Devices). Total pore formation was determined by lysing cells with Triton X-100.

### Lactate dehydrogenase release assay and ELISA

BMDMs were seeded in 24-well plates (5 X 10^5^ cells/well). Infections were performed in RPMI1640 medium without phenol red, 15 mM HEPES and 2 g/l NaHCO_3_ supplemented with 10% FBS. After 8 hours of infection, the supernatants were collected for analysis of lactate dehydrogenase (LDH) release. Total LDH was determined by lysing the cultures with Triton X-100. LDH was quantified using the CytoTox 96 LDH-release kit (Promega). For cytokine determination, enzyme-linked immunosorbent assay (ELISA) were used. BMDMs were seeded into 24-well plates (5 X 10^5^ cells/well) and infected with WT Lp, *fliI*^*-*^ and *flaA*^*-*^ (MOI 10) for 24 hours. BMDMs supernatant was assessed using ELISA kits according to manufacturer´s recommendations (BD Biosciences).

### Ethics statement

The care of the mice was in compliance with the institutional guidelines on ethics in animal experiments; approved by CETEA (Comissão de Ética em Experimentação Animal da Faculdade de Medicina de Ribeirão Preto, approved protocol number 218/2014). CETEA follow the Brazilian national guidelines recommended by CONCEA (Conselho Nacional de Controle em Experimentação o Animal). For euthanasia, the mice were treated with ketamine and xylazine (50 mg/kg and 10 mg/kg, respectively) by intravenous injection.

### Statistical analysis

The data were plotted and analyzed using GraphPad Prism 5.0 software. The statistical significance was calculated using the Student’s t-test or analysis of variance (ANOVA). Nonparametric test Mann–Whitney U test were used for analysis of in vivo experiments. Differences were considered statistically significant when *P* was <0.05, as indicated by an asterisk in the figures.

## Supporting information

S1 Fig Restriction of *L*. *pneumophila* replication is fully dependent on NLRC4 and flagellin, and partially dependent on caspase-1/11. Bone marrow-derived macrophages (BMDMs) from C57BL/6 (open circles), *Nlrc4*^*-/-*^ (closed squares), *Casp1/11*^*-/-*^ (closed triangles) and *Asc*^*-/-*^ (open inverted triangles) mice were infected with *L*. *pneumophila* for CFU determination. (A, B) Cells were infected with wild-type *L*. *pneumophila* (WT Lp). (C, D) Cells were infected with motility-deficient mutants expressing flagellin (*fliI*^-^). (E, F) Cells were infected with flagellin-deficient mutants (*flaA*^*-*^). BMDMs were infected with 3x10^3^ (MOI 0.015) or 2x10^5^ (MOI 10) bacteria per well and incubated for 24, 48, 72 and 96 hours for CFU determination. Data show the average ± SD of triplicate wells. *, *P*<0.05 compared with *Casp1/11*^*-/-*^ BMDMs, ANOVA. Data are presented for one representative experiment of two experiments with similar results.
(TIF)

S2 Fig Caspase-8 but not caspase-3 and caspase-7 colocalize with NLRC4-GFP and ASC-GFP puncta. Bone marrow-derived macrophages (BMDMs) generated from *Casp1/11*^*-/-*^ mice were transduced with retrovirus encoding NLRC4-GFP (A) or ASC-GFP (B) and infected with motility-deficient *L*. *pneumophila* mutants expressing flagellin (*fliI*^-^) at a MOI of 10 for 8 h. (A-B) The percentage of colocalization of caspase-3, caspase-7 and caspase-8 with NLRC4-GFP and ASC-GFP was determined using an epifluorescence microscope. (C-E) BMDMs generated from *Casp1/11*^*-/-*^ mice were infected with *fliI*^-^ at a MOI of 10 for 8 h. The cultures were fixed and stained with anti-ASC (green), anti-caspase-3 (red) (C), anti-caspase-7 (red) (D), anti-caspase-8 (red) (E). Cell nuclei were stained with DAPI (cyan). Images were acquired by multiphoton microscopy with a 63x oil immersion objective and analyzed using ImageJ software. The images are the maximal projection of a z project. Scale bar, 10μm. Data show the average ± SD of triplicate wells. *, *P*<0.05, Student´s t test. Data are presented for one representative experiment of two experiments with similar results.
(TIF)

S3 Fig AIM2 does not colocalize and is not required for NLRC4-GFP puncta formation. Bone marrow-derived macrophages (BMDMs) generated from *Casp1/11*^*-/-*^ and *Aim2/Casp1/11*^*-/-*^ mice were transduced with retrovirus encoding NLRC4-GFP and infected with wild-type *L*. *pneumophila* (WT) or with motility-deficient *L*. *pneumophila* mutants expressing flagellin (*fliI*^-^) at a MOI of 10 for 8 h. (A) The cultures were stained with anti-AIM2 (red), the cell nuclei were stained with DAPI (cyan) and the NLRC4-GFP puncta is shown in green. The percentage of colocalization of AIM2 with NLRC4-GFP is shown. Images were acquired by multiphoton microscopy with a 63x oil immersion objective and analyzed using ImageJ software. Scale bar, 10μm. (B) Quantification of the number of transduced cells containing NLRC4-GFP in response to WT or *fliI*^*-*^ infection was estimated in *Casp1/11*^*-/-*^ and *Aim2/Casp1/11*^*-/-*^ BMDMs. Data show the average ± SD of triplicate wells. NS, not significant, Student´s t test. NI, uninfected. Data are presented for one representative experiment of two experiments with similar results.
(TIF)

S4 Fig AIM2 is not required for caspase-8 activation in response to flagellated *L*. *pneumophila*. Bone marrow-derived macrophages (BMDMs) generated from *Casp1/11*^*-/-*^ and *Aim2/Casp1/11*^*-/-*^ mice were infected with motility-deficient *L*. *pneumophila* mutants expressing flagellin (*fliI*^-^) or with flagellin-deficient bacteria (*flaA*^*-*^) at a MOI of 10 for 8 hours. The activity of caspase-8 was measured using the Caspase-8 Glo Assay. Data show the average ± SD of triplicate wells. *, *P*<0.05, Student´s t test. NI, uninfected. Data are presented for one representative experiment of two experiments with similar results.
(TIF)

S5 Fig Caspase-8 quantification in the western blot shown in Fig 4A. Bone marrow-derived macrophages (BMDMs) generated from *Casp1/11*^*-/-*^ mice were transduced with a retrovirus encoding shRNA sequences to target caspase-8 (Seq1, Seq2) and a non-target shRNA sequence (NT). The silencing was confirmed by western blot analysis (Fig 4A). Cell lysates were separated by SDS-PAGE, blotted and probed with anti-caspase-8 (pro-caspase-8 p55) and anti-α-actin. Immunoblots were analyzed in Image J software and the caspase-8 p55 to α-actin ratio is shown.
(TIF)

S6 Fig AIM2 is not required for NLRC4-mediated restriction of *L*. *pneumophila* replication in macrophages. Bone marrow-derived macrophages (BMDMs) from C57BL/6, *Nlrc4*^*-/-*^, *Casp1/11*^*-/-*^ and *Aim2/Casp1/11*^*-/-*^ mice were infected with motility-deficient *L*. *pneumophila* mutants expressing flagellin (*fliI*^-^) at a MOI of 0.015. The cultures were incubated for 24, 48, 72 and 96 hours after infection for CFU determination. Data show the averages ± SD of triplicate wells. *, *P*<0.05, compared with *Casp1/11*^*-/-*^ cells. Student´s t test. Data are presented for one representative experiment of three experiments with similar results.
(TIF)

S7 Fig Caspase-8 quantification in the western blot shown in Fig 5E. Bone marrow-derived macrophages (BMDMs) generated from *Casp1/11*^*-/-*^ and *Asc/Casp1/11*^*-/-*^ mice were transduced with a retrovirus encoding shRNA sequences to target caspase-8 (Seq1, Seq2) and a non-target shRNA sequence (NT). The silencing was confirmed by western blot analysis (Fig 5E). Cell lysates were separated by SDS-PAGE, blotted and probed with anti-caspase-8 (pro-caspase-8 p55) and anti-α-actin. Immunoblots were analyzed in Image J software and the caspase-8 p55 to α-actin ratio is shown.
(TIF)

S8 Fig AIM2 is not required for NLRC4-mediated restriction of *L*. *pneumophila* infection in vivo. C57BL/6 (open circles), *Nlrc4*^-/-^ (closed squares), *Casp1/11*^-/-^ (closed triangles), *Aim2/Casp1/11*^*-/-*^ (open diamond) and *Asc/Casp1/11*^-/-^ (closed triangles) mice were infected intranasally with 1x10^5^ motility-deficient *L*. *pneumophila* mutants expressing flagellin (*fliI*^-^). The mice were euthanatized at 4 and 48 hours after infection. Dilutions of the lung homogenates were added to charcoal-yeast extract agar plates for colony-forming unit determination. Each dot represents a single animal, and the horizontal lines represent averages. *, *P*<0.05, Student´s t test. NS, not significant.
(TIF)

S9 Fig The NLRC4/ASC/caspase-8 inflammasome is important for pore formation and cell death independently of caspase-1/11. Bone marrow-derived macrophages (BMDMs) were generated from C57BL/6, *Casp1/11*^*-/-*^ and *Asc/Casp1/11*^*-/-*^ mice and infected with motility-deficient *L*. *pneumophila* mutants expressing flagellin (*fliI*^-^) or with flagellin-deficient bacteria (*flaA*^*-*^) at a MOI of 10. (A-C) Pore formation was assessed fluorometrically in real time by the uptake of propidium iodide. RFU (%) represents the percentage of RFU estimated with cells lysed with Triton X-100. (D) After 8 hours the LDH release was measured using the CytoTox 96 LDH-release kit. The LDH release (%) represents the percentage of LDH released compared with cells lysed with Triton X-100. Data show the average ± SD of triplicate wells. *, *P*<0.05, Student´s t test. NS, not significant; RFU, relative fluorescence units; NI, uninfected. Data are presented for one representative experiment of five (A-C) and two (D) experiments with similar results.
(TIF)

S10 Fig IL-1β is not efficiently produced by *Casp1/11*^*-/-*^ and *Asc/Casp1/11*^*-/-*^ macrophages. Bone marrow-derived macrophages (BMDMs) were generated from C57BL/6, *Casp1/11*^*-/-*^ and *Asc/Casp1/11*^*-/-*^ mice and infected with wild-type *L*. *pneumophila* (WT Lp), motility-deficient mutants expressing flagellin (*fliI*^-^) or with flagellin-deficient mutants (*flaA*^*-*^) at a MOI of 10. The production of IL-1β (A) and IL-12p40 (B) in the tissue culture supernatants was estimated by ELISA at 24 hours after infection. Data show the average ± SD of triplicate wells. *, *P*<0.05, Student´s t test. nd, not detected; RFU, relative fluorescence units; NI, uninfected. Data are presented for one representative experiment of two experiments with similar results.
(TIF)

S11 Fig Gasdermin-D is important for pore formation in C57BL/6 but not in *Casp1/11*^*-/-*^ macrophages. BMDMs generated from C57BL/6 (A-D) and *Casp1/11*^*-/-*^ (E-H) mice were transduced with a retrovirus encoding shRNA sequence to target Gasdermin D (GSDMD) (Seq1) and a non-target shRNA sequence (NT). Transduced cells were infected with wild-type *L*. *pneumophila* (WT Lp) (B and F), motility-deficient mutants expressing flagellin (*fliI*^-^) (C and G) or with flagellin-deficient mutants (*flaA*^*-*^) (D and H) at a MOI of 10. Pore formation was assessed fluorometrically in real time by the uptake of propidium iodide. The RFU (%) represents the percentage of RFU compared with cells lysed with Triton X-100. Data show the average ± SD of triplicate wells. RFU, relative fluorescence units; NI, uninfected. Data are presented for one representative experiment of two experiments with similar results.
(TIF)
