# Supplementary figures and images for: Inhibition of caspase-1 or gasdermin-D enable caspase-8 activation in the Naip5/NLRC4/ASC inflammasome
Source: PLoS Pathog. 2017 Aug 3;13(8):e1006502. doi: 10.1371/journal.ppat.1006502 (PMC5542441; doi:10.1371/journal.ppat.1006502)

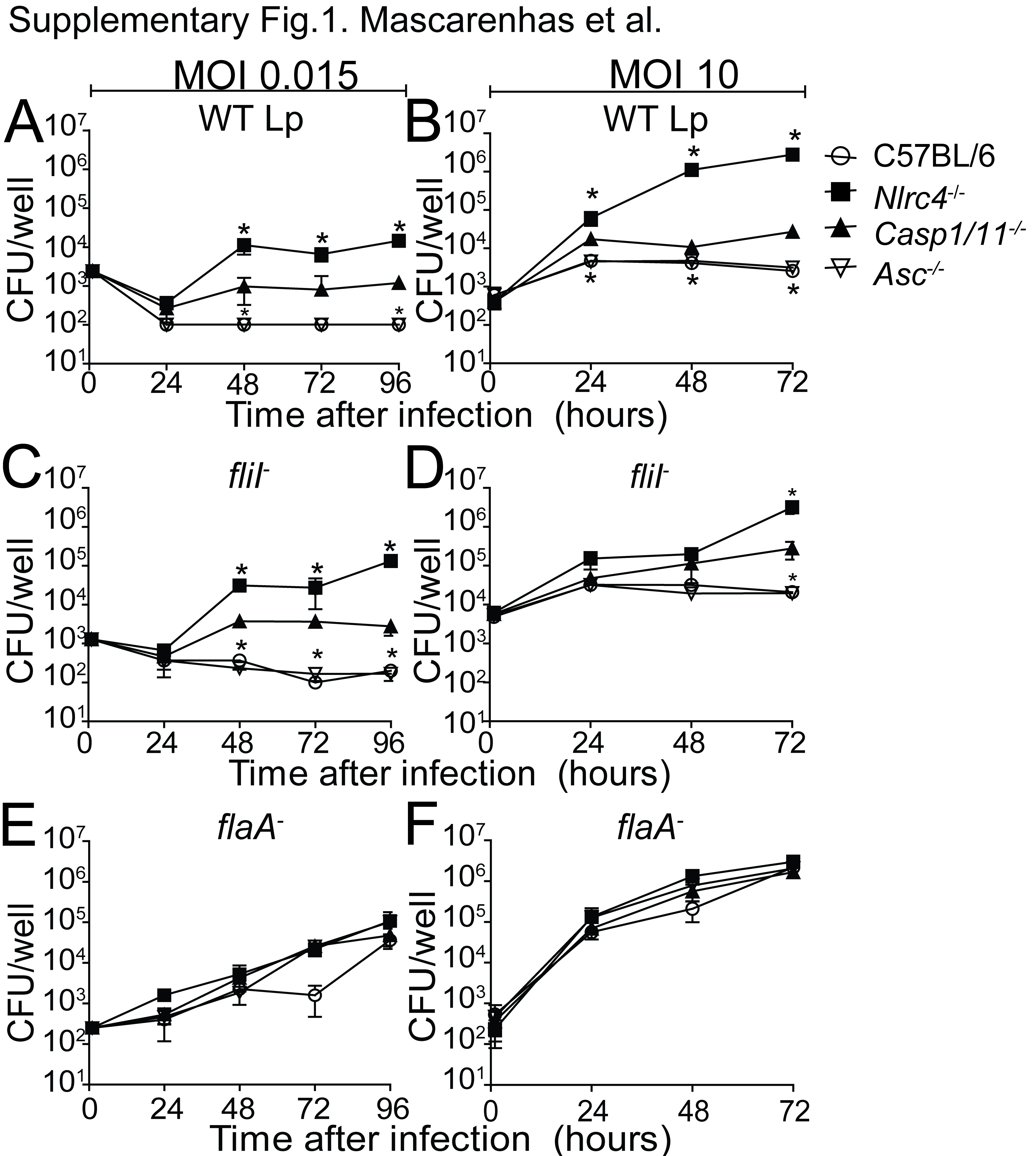

Supplement: S1 Fig — Bone marrow-derived macrophages (BMDMs) from C57BL/6 (open circles), Nlrc4-/- (closed squares), Casp1/11-/- (closed triangles) and Asc-/- (open inverted triangles) mice were infected with L. pneumophila for CFU determination. (A, B) Cells were infected with wild-type L. pneumophila (WT Lp). (C, D) Cells were infected with motility-deficient mutants expressing flagellin (fliI-). (E, F) Cells were infected with flagellin-deficient mutants (flaA-). BMDMs were infected with 3x10^3^ (MOI 0.015) or 2x10^5^ (MOI 10) bacteria per well and incubated for 24, 48, 72 and 96 hours for CFU determination. Data show the average ± SD of triplicate wells. *, P<0.05 compared with Casp1/11-/- BMDMs, ANOVA. Data are presented for one representative experiment of two experiments with similar results. (TIF) [file ppat.1006502.s001.tif]

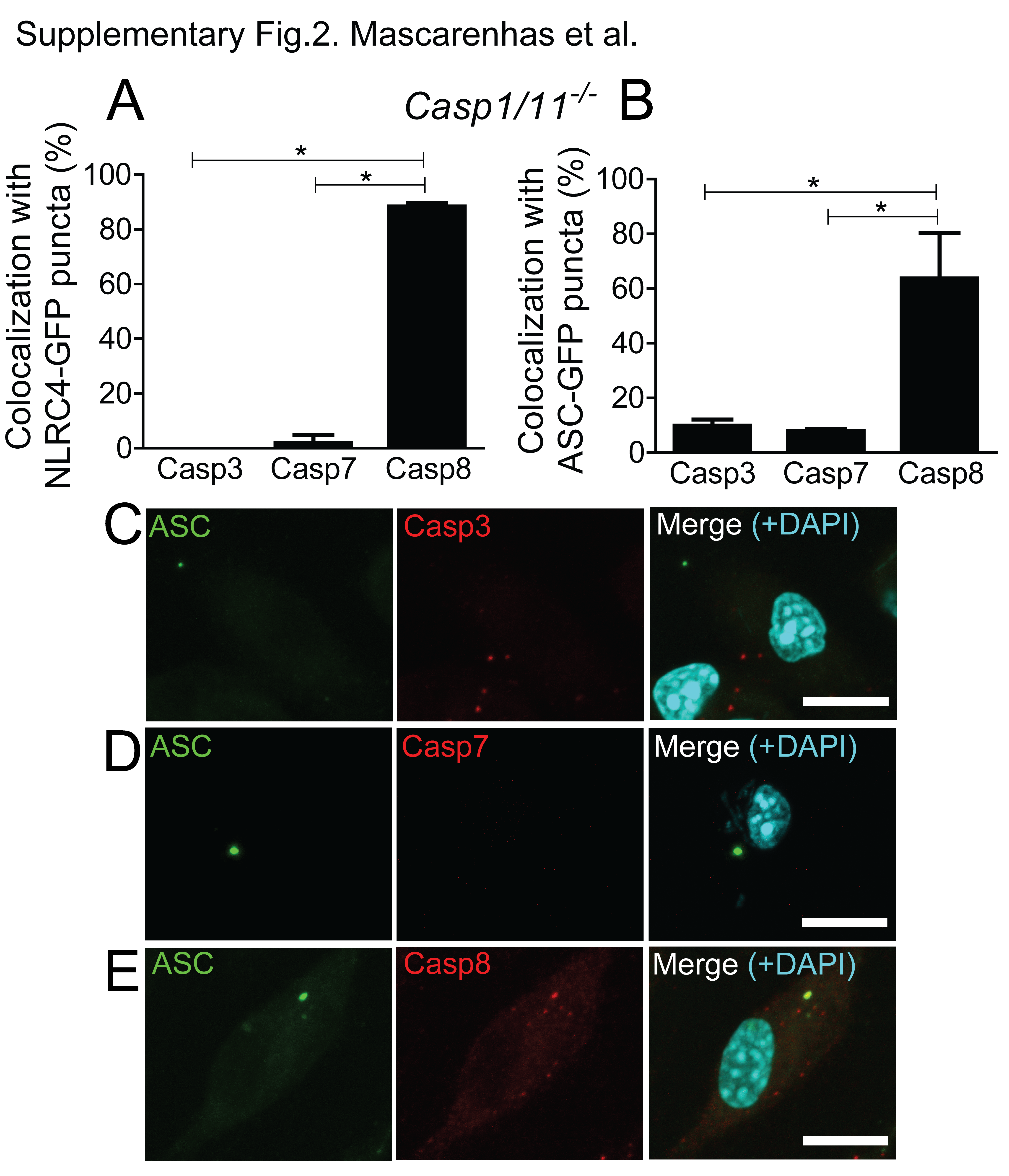

Supplement: S2 Fig — Bone marrow-derived macrophages (BMDMs) generated from Casp1/11-/- mice were transduced with retrovirus encoding NLRC4-GFP (A) or ASC-GFP (B) and infected with motility-deficient L. pneumophila mutants expressing flagellin (fliI-) at a MOI of 10 for 8 h. (A-B) The percentage of colocalization of caspase-3, caspase-7 and caspase-8 with NLRC4-GFP and ASC-GFP was determined using an epifluorescence microscope. (C-E) BMDMs generated from Casp1/11-/- mice were infected with fliI- at a MOI of 10 for 8 h. The cultures were fixed and stained with anti-ASC (green), anti-caspase-3 (red) (C), anti-caspase-7 (red) (D), anti-caspase-8 (red) (E). Cell nuclei were stained with DAPI (cyan). Images were acquired by multiphoton microscopy with a 63x oil immersion objective and analyzed using ImageJ software. The images are the maximal projection of a z project. Scale bar, 10μm. Data show the average ± SD of triplicate wells. *, P<0.05, Student´s t test. Data are presented for one representative experiment of two experiments with similar results. (TIF) [file ppat.1006502.s002.tif]

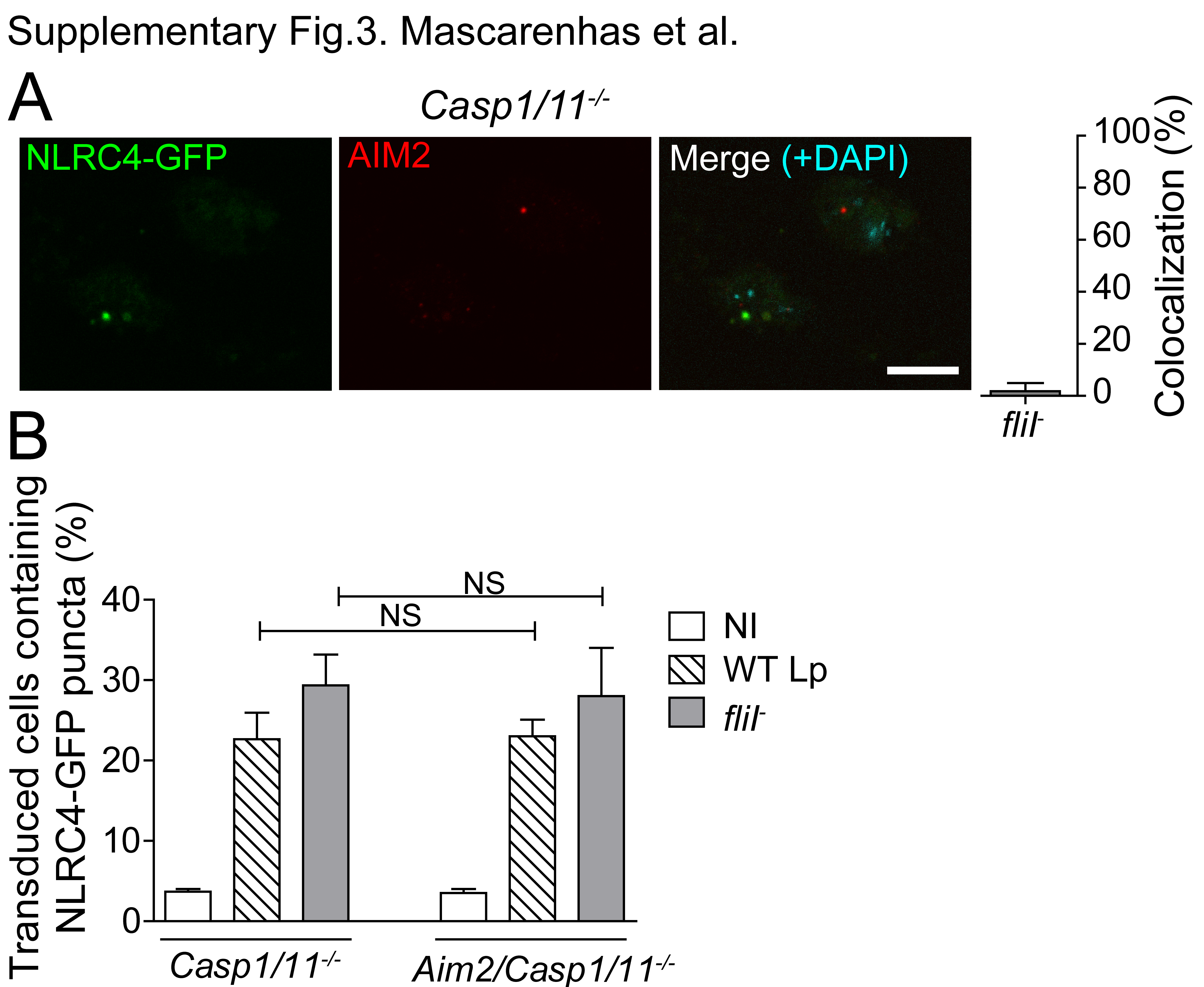

Supplement: S3 Fig — Bone marrow-derived macrophages (BMDMs) generated from Casp1/11-/- and Aim2/Casp1/11-/- mice were transduced with retrovirus encoding NLRC4-GFP and infected with wild-type L. pneumophila (WT) or with motility-deficient L. pneumophila mutants expressing flagellin (fliI-) at a MOI of 10 for 8 h. (A) The cultures were stained with anti-AIM2 (red), the cell nuclei were stained with DAPI (cyan) and the NLRC4-GFP puncta is shown in green. The percentage of colocalization of AIM2 with NLRC4-GFP is shown. Images were acquired by multiphoton microscopy with a 63x oil immersion objective and analyzed using ImageJ software. Scale bar, 10μm. (B) Quantification of the number of transduced cells containing NLRC4-GFP in response to WT or fliI- infection was estimated in Casp1/11-/- and Aim2/Casp1/11-/- BMDMs. Data show the average ± SD of triplicate wells. NS, not significant, Student´s t test. NI, uninfected. Data are presented for one representative experiment of two experiments with similar results. (TIF) [file ppat.1006502.s003.tif]

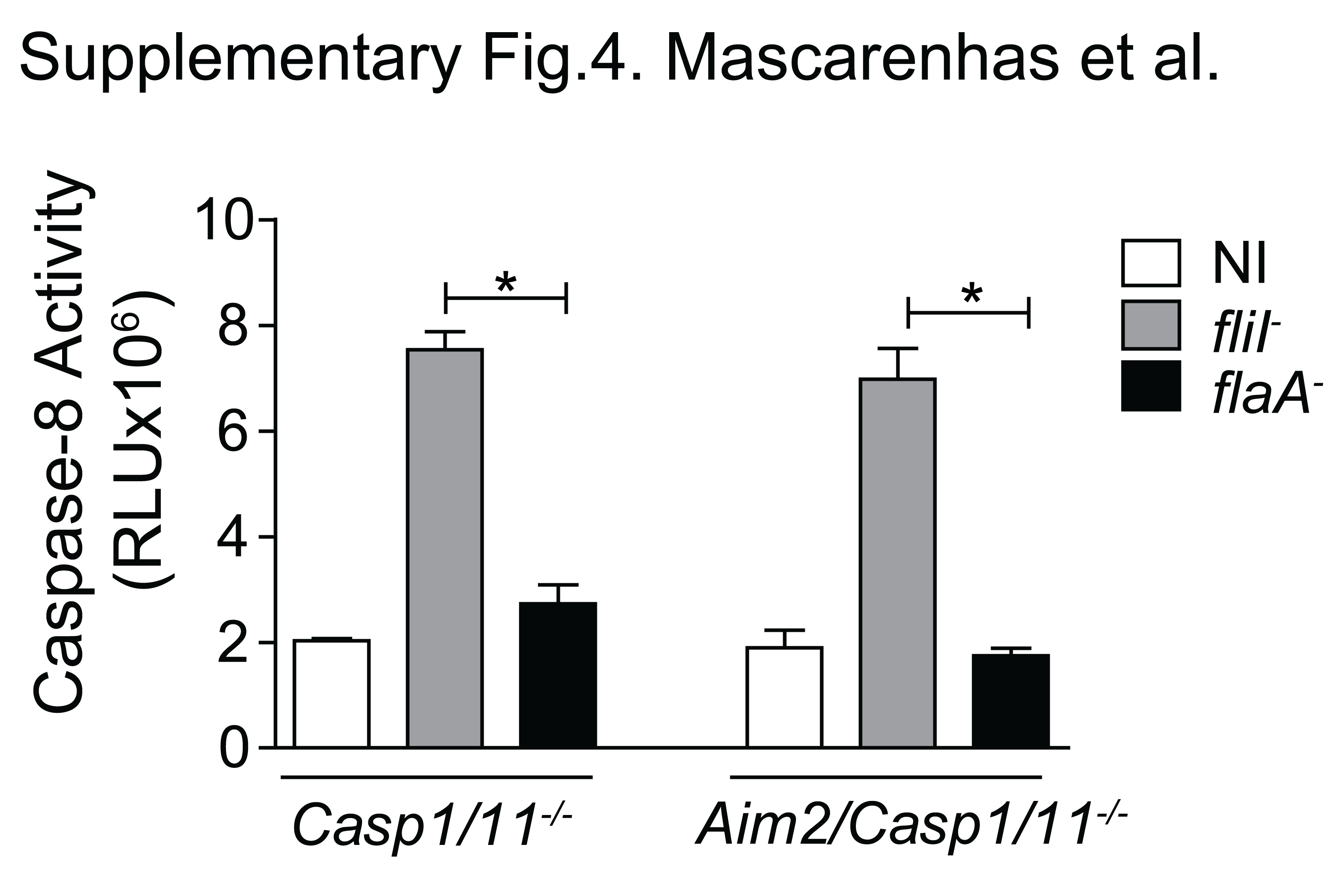

Supplement: S4 Fig — Bone marrow-derived macrophages (BMDMs) generated from Casp1/11-/- and Aim2/Casp1/11-/- mice were infected with motility-deficient L. pneumophila mutants expressing flagellin (fliI-) or with flagellin-deficient bacteria (flaA-) at a MOI of 10 for 8 hours. The activity of caspase-8 was measured using the Caspase-8 Glo Assay. Data show the average ± SD of triplicate wells. *, P<0.05, Student´s t test. NI, uninfected. Data are presented for one representative experiment of two experiments with similar results. (TIF) [file ppat.1006502.s004.tif]

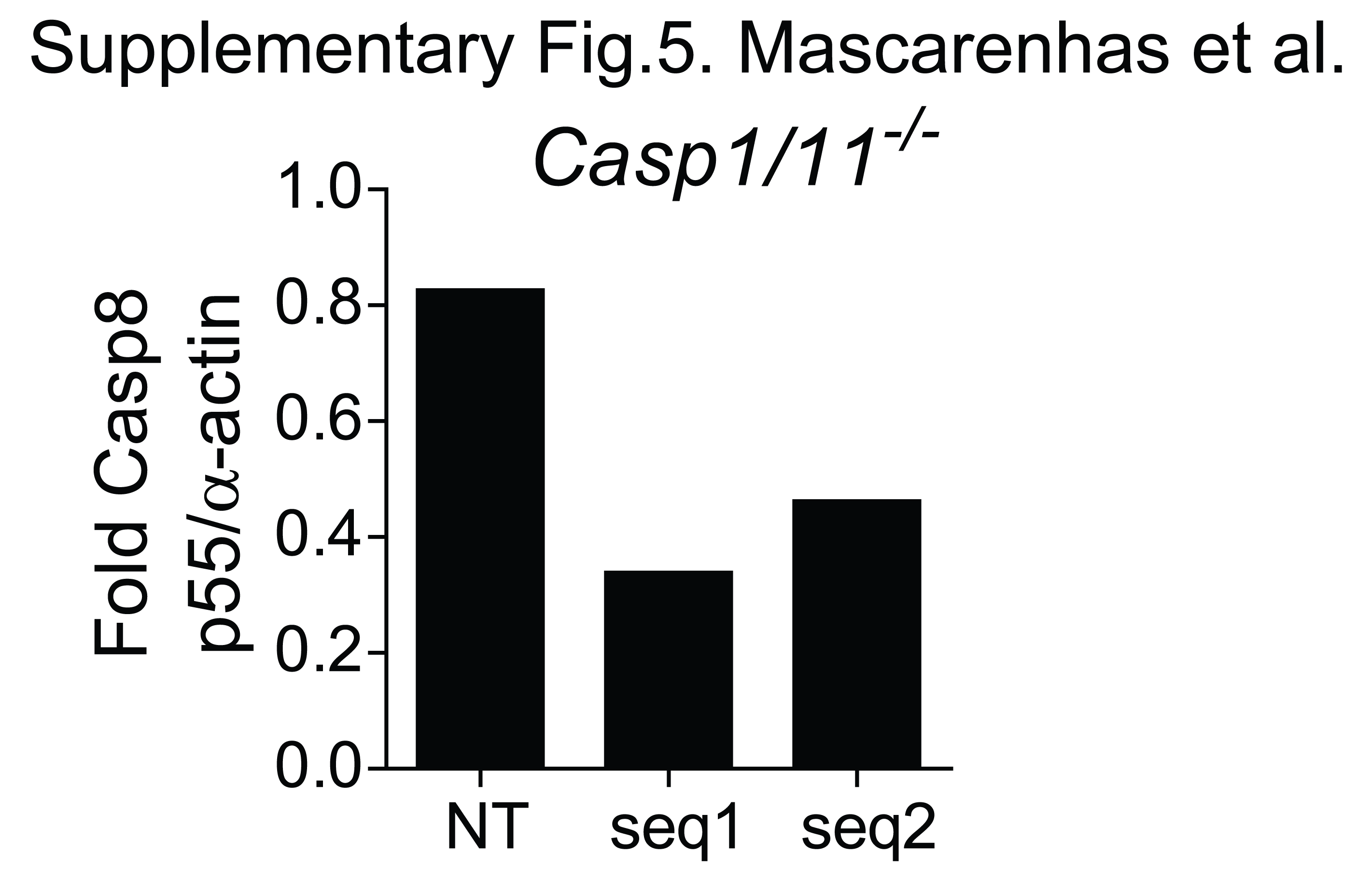

Supplement: S5 Fig — Bone marrow-derived macrophages (BMDMs) generated from Casp1/11-/- mice were transduced with a retrovirus encoding shRNA sequences to target caspase-8 (Seq1, Seq2) and a non-target shRNA sequence (NT). The silencing was confirmed by western blot analysis (Fig 4A). Cell lysates were separated by SDS-PAGE, blotted and probed with anti-caspase-8 (pro-caspase-8 p55) and anti-α-actin. Immunoblots were analyzed in Image J software and the caspase-8 p55 to α-actin ratio is shown. (TIF) [file ppat.1006502.s005.tif]

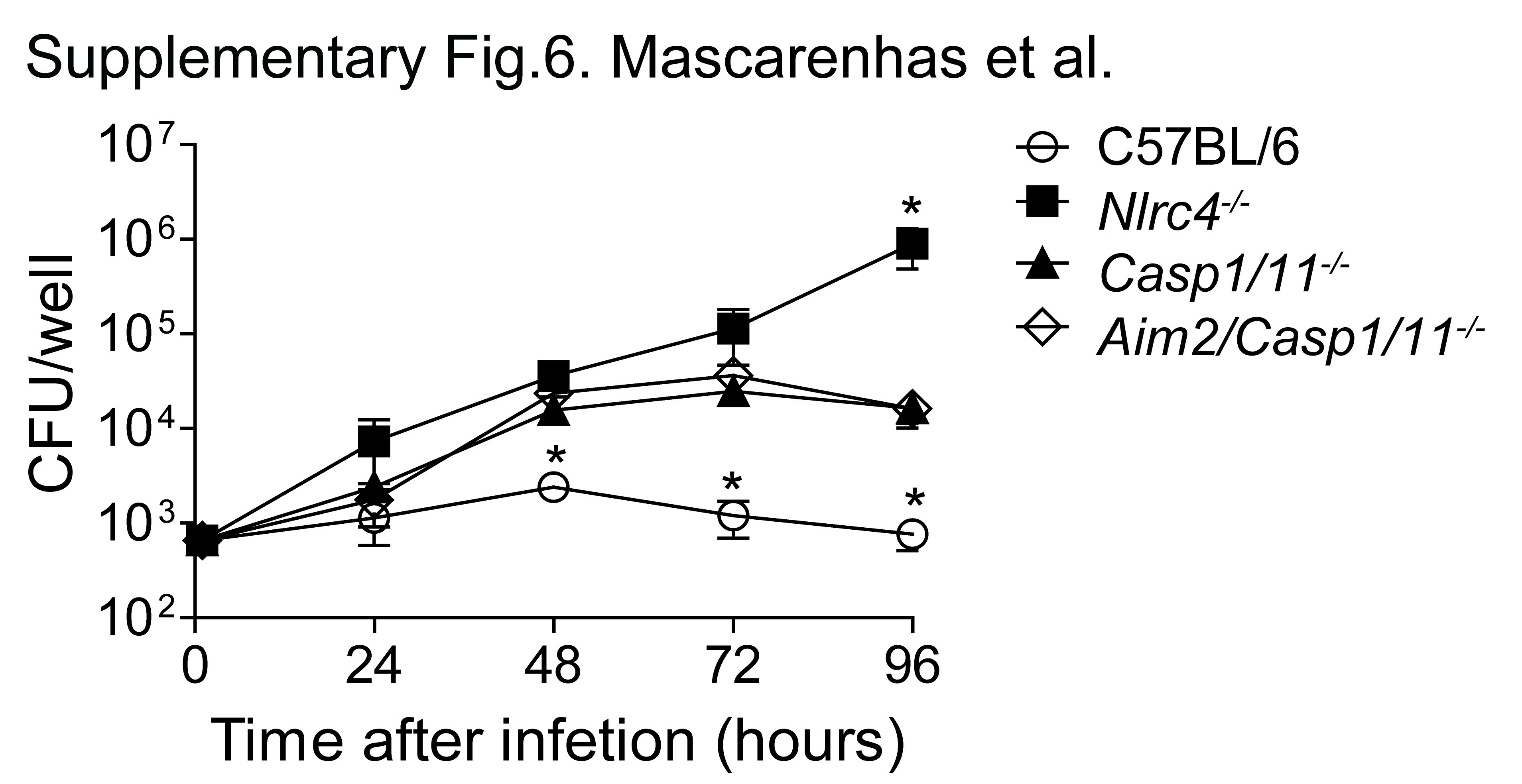

Supplement: S6 Fig — Bone marrow-derived macrophages (BMDMs) from C57BL/6, Nlrc4-/-, Casp1/11-/- and Aim2/Casp1/11-/- mice were infected with motility-deficient L. pneumophila mutants expressing flagellin (fliI-) at a MOI of 0.015. The cultures were incubated for 24, 48, 72 and 96 hours after infection for CFU determination. Data show the averages ± SD of triplicate wells. *, P<0.05, compared with Casp1/11-/- cells. Student´s t test. Data are presented for one representative experiment of three experiments with similar results. (TIF) [file ppat.1006502.s006.tif]

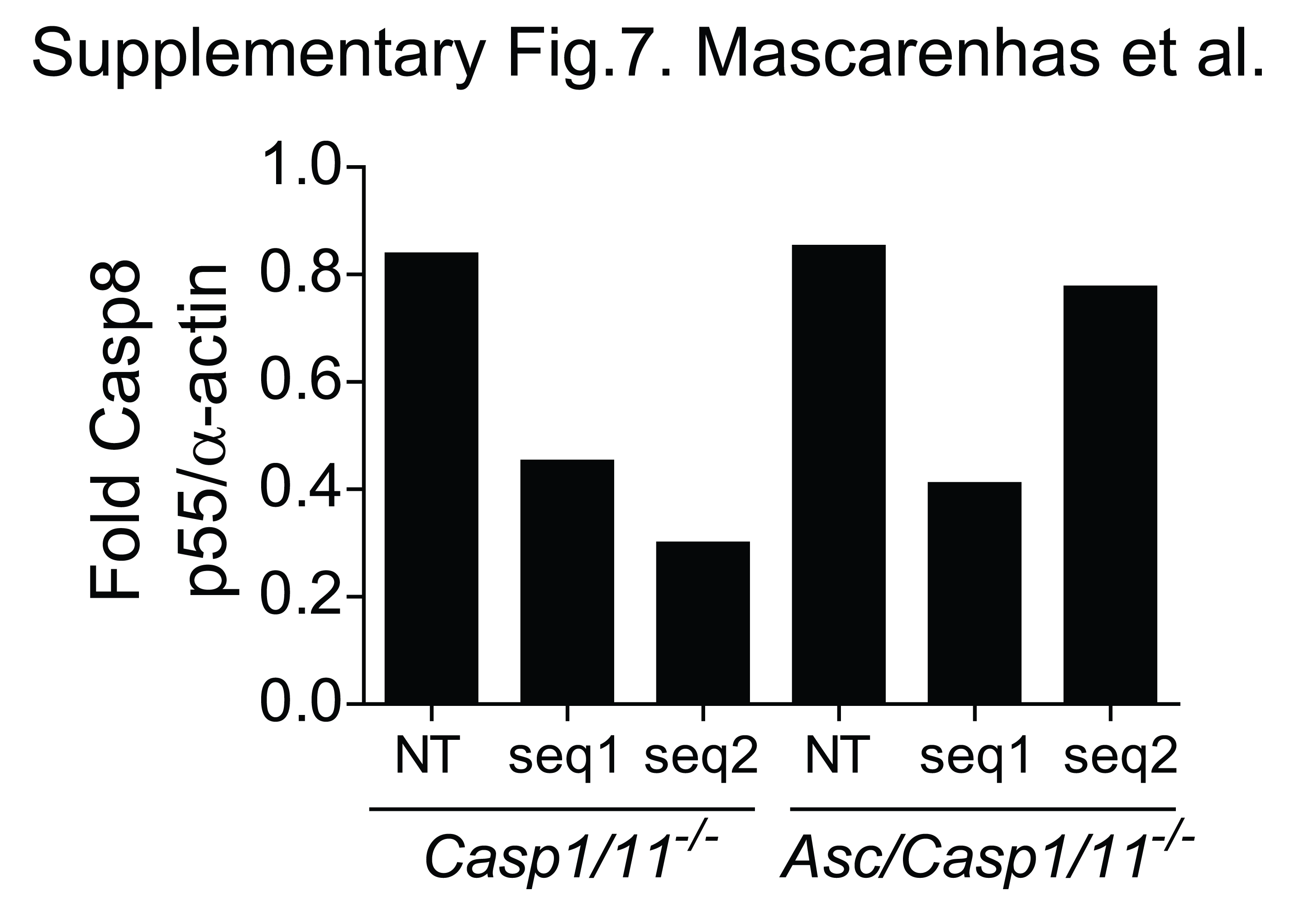

Supplement: S7 Fig — Bone marrow-derived macrophages (BMDMs) generated from Casp1/11-/- and Asc/Casp1/11-/- mice were transduced with a retrovirus encoding shRNA sequences to target caspase-8 (Seq1, Seq2) and a non-target shRNA sequence (NT). The silencing was confirmed by western blot analysis (Fig 5E). Cell lysates were separated by SDS-PAGE, blotted and probed with anti-caspase-8 (pro-caspase-8 p55) and anti-α-actin. Immunoblots were analyzed in Image J software and the caspase-8 p55 to α-actin ratio is shown. (TIF) [file ppat.1006502.s007.tif]

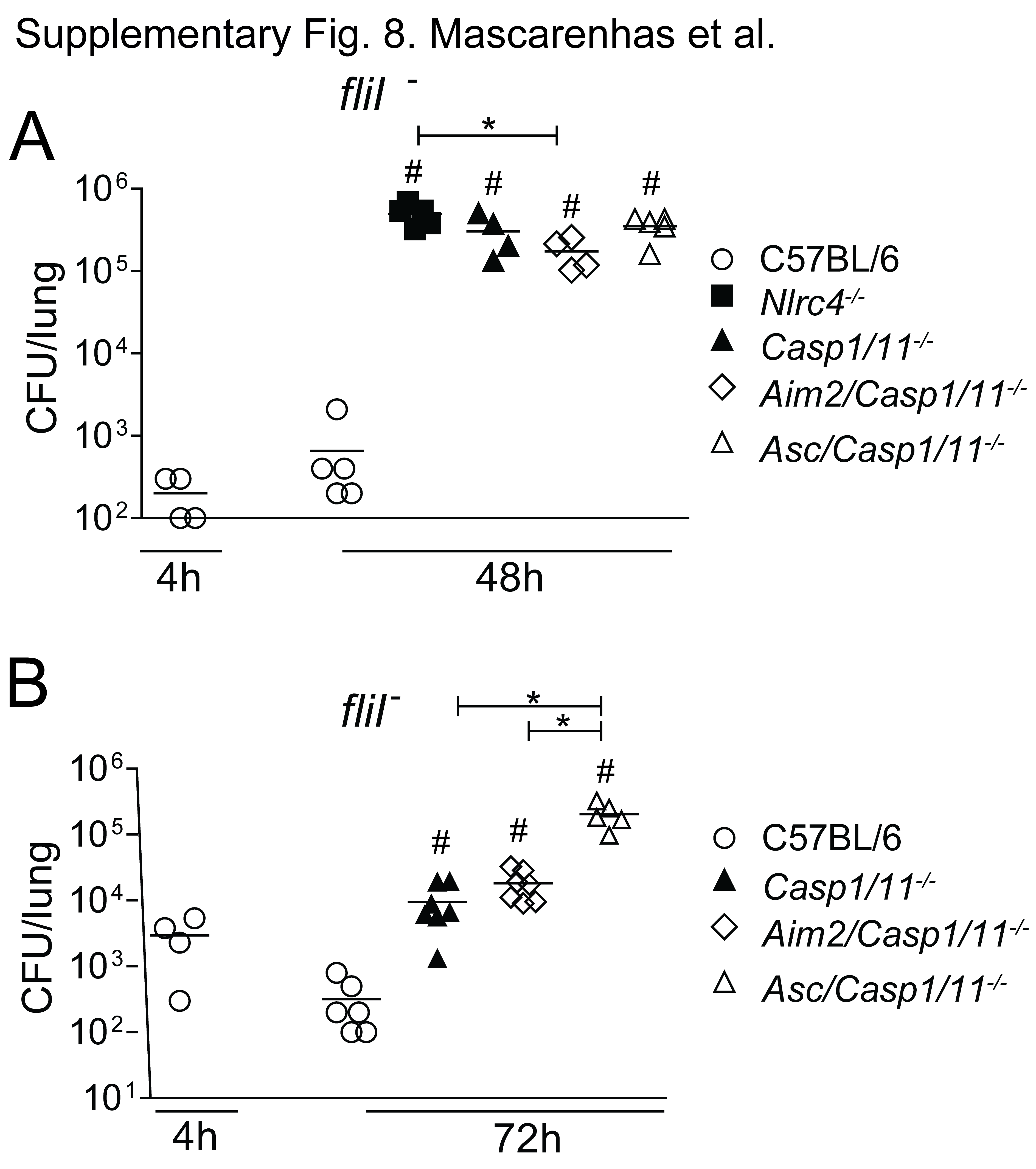

Supplement: S8 Fig — C57BL/6 (open circles), Nlrc4-/- (closed squares), Casp1/11-/- (closed triangles), Aim2/Casp1/11-/- (open diamond) and Asc/Casp1/11-/- (closed triangles) mice were infected intranasally with 1x10^5^ motility-deficient L. pneumophila mutants expressing flagellin (fliI-). The mice were euthanatized at 4 and 48 hours after infection. Dilutions of the lung homogenates were added to charcoal-yeast extract agar plates for colony-forming unit determination. Each dot represents a single animal, and the horizontal lines represent averages. *, P<0.05, Student´s t test. NS, not significant. (TIF) [file ppat.1006502.s008.tif]

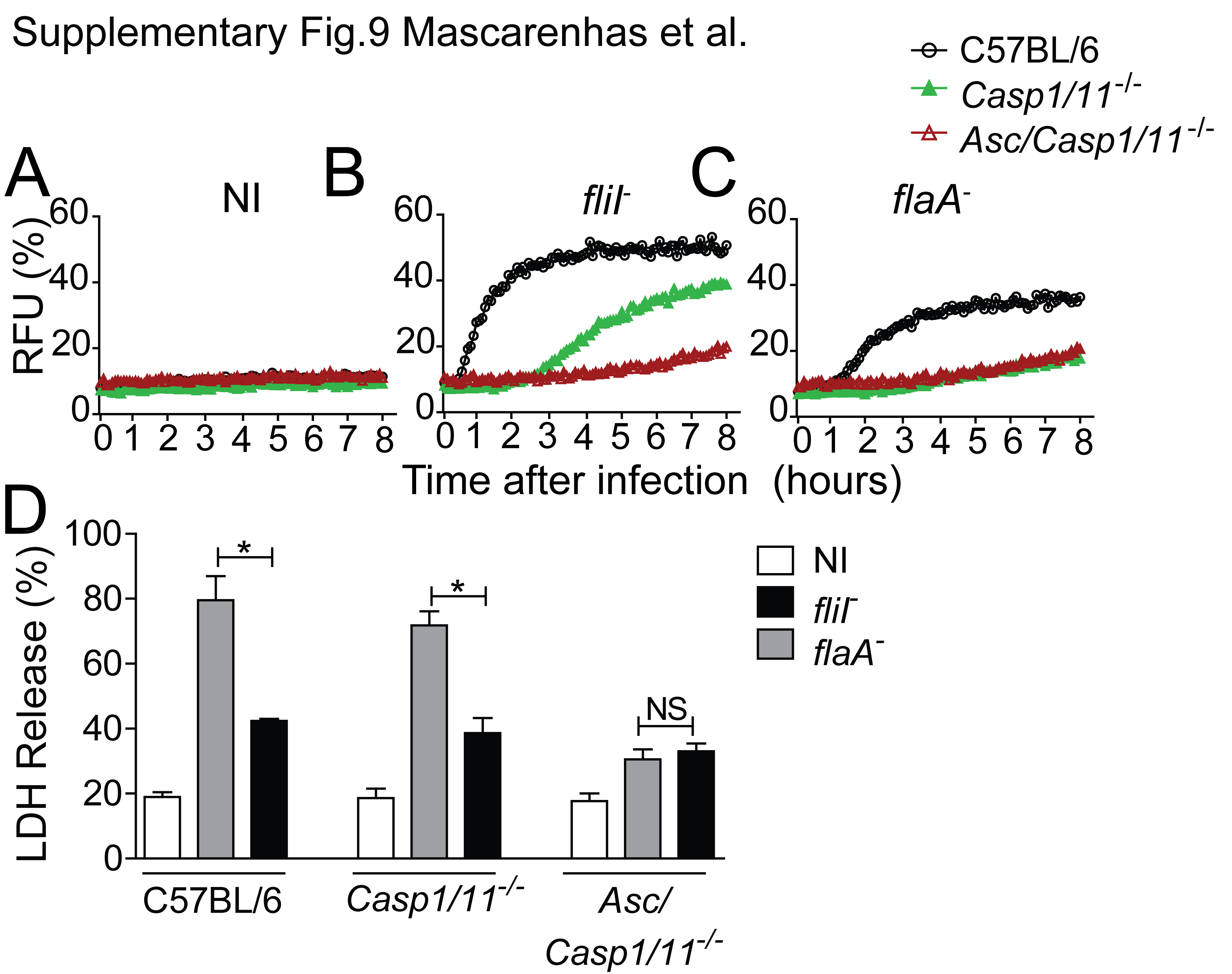

Supplement: S9 Fig — Bone marrow-derived macrophages (BMDMs) were generated from C57BL/6, Casp1/11-/- and Asc/Casp1/11-/- mice and infected with motility-deficient L. pneumophila mutants expressing flagellin (fliI-) or with flagellin-deficient bacteria (flaA-) at a MOI of 10. (A-C) Pore formation was assessed fluorometrically in real time by the uptake of propidium iodide. RFU (%) represents the percentage of RFU estimated with cells lysed with Triton X-100. (D) After 8 hours the LDH release was measured using the CytoTox 96 LDH-release kit. The LDH release (%) represents the percentage of LDH released compared with cells lysed with Triton X-100. Data show the average ± SD of triplicate wells. *, P<0.05, Student´s t test. NS, not significant; RFU, relative fluorescence units; NI, uninfected. Data are presented for one representative experiment of five (A-C) and two (D) experiments with similar results. (TIF) [file ppat.1006502.s009.tif]

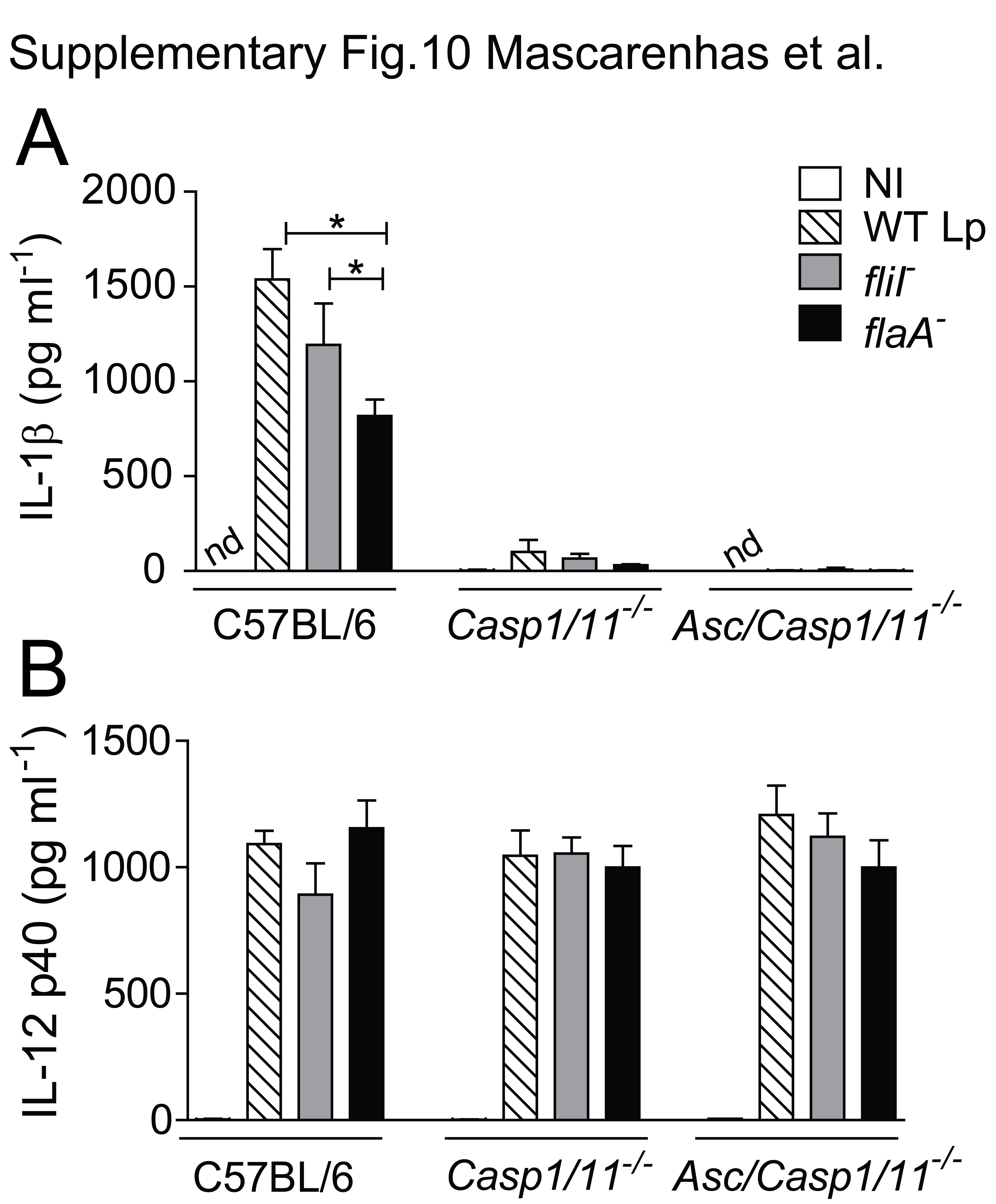

Supplement: S10 Fig — Bone marrow-derived macrophages (BMDMs) were generated from C57BL/6, Casp1/11-/- and Asc/Casp1/11-/- mice and infected with wild-type L. pneumophila (WT Lp), motility-deficient mutants expressing flagellin (fliI-) or with flagellin-deficient mutants (flaA-) at a MOI of 10. The production of IL-1β (A) and IL-12p40 (B) in the tissue culture supernatants was estimated by ELISA at 24 hours after infection. Data show the average ± SD of triplicate wells. *, P<0.05, Student´s t test. nd, not detected; RFU, relative fluorescence units; NI, uninfected. Data are presented for one representative experiment of two experiments with similar results. (TIF) [file ppat.1006502.s010.tif]

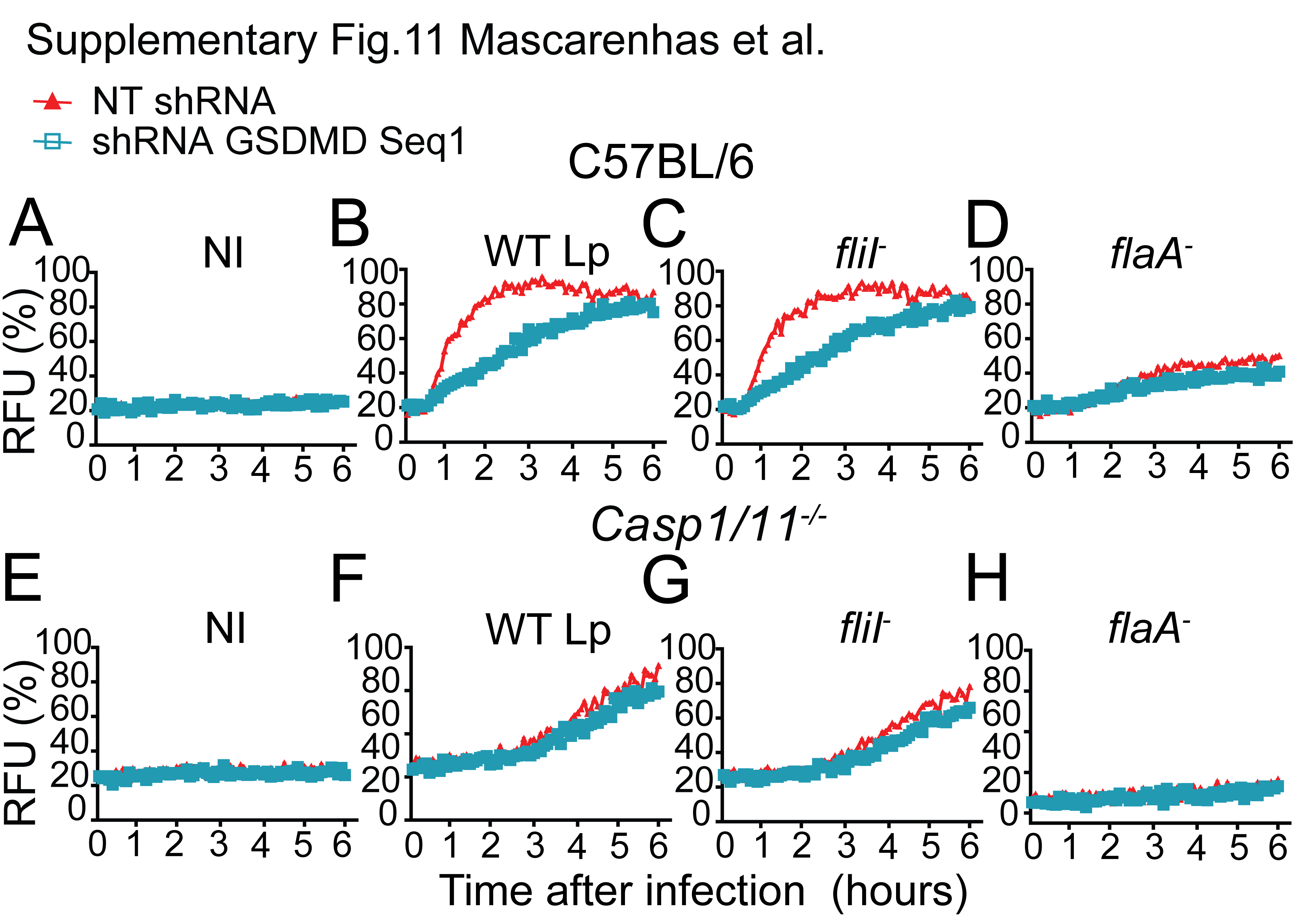

Supplement: S11 Fig — BMDMs generated from C57BL/6 (A-D) and Casp1/11-/- (E-H) mice were transduced with a retrovirus encoding shRNA sequence to target Gasdermin D (GSDMD) (Seq1) and a non-target shRNA sequence (NT). Transduced cells were infected with wild-type L. pneumophila (WT Lp) (B and F), motility-deficient mutants expressing flagellin (fliI-) (C and G) or with flagellin-deficient mutants (flaA-) (D and H) at a MOI of 10. Pore formation was assessed fluorometrically in real time by the uptake of propidium iodide. The RFU (%) represents the percentage of RFU compared with cells lysed with Triton X-100. Data show the average ± SD of triplicate wells. RFU, relative fluorescence units; NI, uninfected. Data are presented for one representative experiment of two experiments with similar results. (TIF) [file ppat.1006502.s011.tif]
